# Low intensity repetitive transcranial magnetic stimulation enhances remyelination by newborn and surviving oligodendrocytes in the cuprizone model of toxic demyelination

**DOI:** 10.1007/s00018-024-05391-0

**Published:** 2024-08-12

**Authors:** Phuong Tram Nguyen, Kalina Makowiecki, Thomas S. Lewis, Alastair J. Fortune, Mackenzie Clutterbuck, Laura A. Reale, Bruce V. Taylor, Jennifer Rodger, Carlie L. Cullen, Kaylene M. Young

**Affiliations:** 1 Menzies Institute for Medical Research, University of Tasmania, Hobart, TAS Australia; 2 School of Biological Sciences, The University of Western Australia, Crawley, WA Australia; 3 Perron Institute for Neurological and Translational Science, Nedlands, WA Australia; 4 Mater Research Institute, The University of Queensland, Woolloongabba, QLD Australia

**Keywords:** Myelin, OPC, NG2, Parvalbumin, PV, cFos, Internode, Cortex, Corpus callosum, rTMS, OLIG2, ASPA

## Abstract

In people with multiple sclerosis (MS), newborn and surviving oligodendrocytes (OLs) can contribute to remyelination, however, current therapies are unable to enhance or sustain endogenous repair. Low intensity repetitive transcranial magnetic stimulation (LI-rTMS), delivered as an intermittent theta burst stimulation (iTBS), increases the survival and maturation of newborn OLs in the healthy adult mouse cortex, but it is unclear whether LI-rTMS can promote remyelination. To examine this possibility, we fluorescently labelled oligodendrocyte progenitor cells (OPCs; *Pdgfrα-CreER* transgenic mice) or mature OLs (*Plp-CreER* transgenic mice) in the adult mouse brain and traced the fate of each cell population over time. Daily sessions of iTBS (600 pulses; 120 mT), delivered during cuprizone (CPZ) feeding, did not alter new or pre-existing OL survival but increased the number of myelin internodes elaborated by new OLs in the primary motor cortex (M1). This resulted in each new M1 OL producing ~ 471 µm more myelin. When LI-rTMS was delivered after CPZ withdrawal (during remyelination), it significantly increased the length of the internodes elaborated by new M1 and callosal OLs, increased the number of surviving OLs that supported internodes in the corpus callosum (CC), and increased the proportion of axons that were myelinated. The ability of LI-rTMS to modify cortical neuronal activity and the behaviour of new and surviving OLs, suggests that it may be a suitable adjunct intervention to enhance remyelination in people with MS.

**Supplementary Information:**

The online version contains supplementary material available at 10.1007/s00018-024-05391-0.

## Introduction

Oligodendrocytes (OLs) elaborate and wrap myelin around axons in the central nervous system (CNS), increasing the resistance and decreasing the capacitance of the axonal membrane, and enabling the saltatory conduction of action potentials [1]. OLs also detect neuronal activity [2, 3] and provide metabolic support to the axons they myelinate [4]. OL loss and demyelination contribute to neurodegeneration and disability accrual in people with multiple sclerosis (MS) [5]. In mice and humans, oligodendrogenesis and spontaneous remyelination can occur in response to a demyelinating injury [6–9]. In people with MS, the level of endogenous remyelination is highly variable and its failure has been attributed to the inability of: (i) OPCs to differentiate into new myelinating OLs within the lesion environment [10, 11], and (ii) surviving OLs to generate new myelin sheaths [9, 12]. Therapeutic approaches designed to enhance remyelination are most likely to be effective if they simultaneously promote repair by new and surviving OLs.

Neuronal activity is a major extrinsic regulator of myelination [13–15]. Repetitive transcranial magnetic stimulation (rTMS) applies focal magnetic fields to induce electric currents in the brain and can be used to noninvasively modulate neuronal activity, with the outcome being influenced by the stimulation intensity, frequency, pattern and number of sessions [16, 17]. rTMS has been delivered to people with MS to evaluate its safety [18, 19] and effect on hand dexterity [20, 21], lower urinary tract function [22, 23], working memory [24], spasticity [25–27] or fatigue [28]. However, these clinical studies did not explore the impact of rTMS on remyelination. We previously reported that 28 consecutive daily sessions of low-intensity (LI)-rTMS, patterned as an intermittent theta burst stimulation (iTBS), increased the survival and maturation of new OLs. LI-rTMS not only increased the number of new OLs in the adult mouse cortex but increased the length of the internodes elaborated [29]. There is some evidence that rTMS can protect against demyelination or enhance remyelination, as 14 consecutive daily sessions of 10 Hz rTMS (0.4 T) reduced the level of gross demyelination detected following a spinal cord injury [30], and 7 consecutive daily sessions of 5 Hz rTMS (1.26 T) reduced the level of hippocampal, cortical and striatal demyelination detected after cuprizone (CPZ)-feeding [31].

To determine whether LI-rTMS can influence the behaviour of new and/or surviving OLs in the demyelinating or remyelinating CNS, we performed cre-lox lineage tracing to follow the fate of OPCs and the new OLs they generate (*Pdgfrα-CreER* transgenic mice), or the mature myelinating OLs that survive demyelination (*Plp-CreER* transgenic mice) in the adult mouse brain. When LI-rTMS was delivered as an iTBS during CPZ feeding, new primary motor cortex (M1) OLs elaborated significantly more internodes than those generated in sham-stimulated mice. Furthermore, when iTBS was delivered after CPZ withdrawal, it increased the length of internodes generated by new M1 OLs and increased the proportion of surviving callosal OLs that contributed to remyelination, resulting in an increased proportion of myelinated axons.

## Materials and methods

### Transgenic mouse genotyping and tamoxifen delivery

All animal experiments were approved by the University of Tasmania Animal Ethics Committee (A0018606 and 28221) and carried out in accordance with the Australian code of practice for the care and use of animals for scientific purposes. *Pdgfrα-CreER™* (RRID: IMSR_JAX:018280), *Plp-CreER*^*T2*^ (RRID: IMSR_JAX:005975), *Rosa26-YFP* (RRID: IMSR_JAX:006148), and *Tau-mGFP* (RRID: IMSR_JAX:021162) mouse lines were purchased from the Jackson Laboratories. *Pdgfrα-CreERT*^*T2*^ transgenic mice [32] were a kind gift from Prof. William D Richardson (University College London). All transgenic mice were maintained on a C56BL/6J background.

To fluorescently label and trace OPCs and the new OLs they produce, heterozygous *Pdgfrα-CreER™* transgenic mice [33] were crossed with homozygous *Rosa26-YFP* Cre-sensitive reporter mice [34] or heterozygous *Pdgfrα-CreER*^*T2*^ transgenic mice [32] were crossed with heterozygous *Tau-lox-STOP-lox-mGFP-IRES-NLS-LacZ-pA* (*Tau-mGFP*) Cre-sensitive reporter mice [35] to generate double heterozygous offspring. To fluorescently label and trace already mature OLs, heterozygous *Plp-CreER*^*T2*^ transgenic mice [36] were crossed with homozygous *Rosa26-YFP* or heterozygous *Tau-mGFP* Cre-sensitive reporter mice to generate double heterozygous offspring. The expression of *Cre*, *Rosa26-YFP* or *mGFP* transgenes was confirmed by polymerase chain reaction (PCR) as described [29]. In brief, genomic DNA (gDNA) was extracted from ear biopsies by ethanol precipitation and PCR performed using 50–100 ng of gDNA with the following primer combinations: Cre 5′ CAGGT CTCAG GAGCT ATGTC CAATT TACTG ACCGTA and Cre 3′ GGTGT TATAAG CAATCC CCAGAA; Rosa26 wildtype 5′ AAAGT CGCTC TGAGT TGTTAT, Rosa26 wildtype 3′ GGAGC GGGAG AAATG GATATG and Rosa26 YFP 5′ GCGAA GAGTT TGTCC TCAACC; GFP 5′ CCCTG AAGTTC ATCTG CACCAC and GFP 3′ TTCTC GTTGG GGTCT TTGCTC. Alternatively, mice were genotyped by quantitative PCR (Transnetyx).

Experimental mice were group-housed with same-sex littermates (2–5 per cage) in Optimice microisolator cages with Bed-o'Cobs 1/4" bedding (Tecniplast) on a 12 h light/dark cycle (lights on 7:00, lights off 19:00) at 21 ± 2 °C with ad libitum access to standard rodent chow (Barrastoc rat and mouse pellets) and water. Mice weighed 16–24 g at the start of the experiment, when female and male mice were randomly assigned to each treatment. Care was taken to ensure littermates and sexes were represented across treatment groups. To initiate Cre-mediated recombination of a reporter transgene, tamoxifen (Tx; Sigma, T5648) was dissolved in corn oil (Sigma, C8267) at a concentration of 40 mg/mL by sonication at 21 °C for 2 h and administered to P60 mice (P60 ± 5 days) by oral gavage at a dose of 300 mg/kg body weight daily for 4 consecutive days.

### CPZ-induced demyelination and remyelination

A diet containing 0.2% (w/w) CPZ (Sigma, C9012) was fed to mice for 4 or 5 weeks, as specified, from P67 (P67 ± 5 days). CPZ was thoroughly mixed into ground rodent chow and 1 mL of H_2_O added per 2 g dry weight. Approximately 25 g of hydrated CPZ chow was prepared per mouse, replaced every second day. Mice were transferred to clean home cages every 4 days to prevent them from ingesting old CPZ chow.

### LI-rTMS

LI-rTMS was delivered as previously described [29] using a custom made 120mT circular coil designed for rodent stimulation (8 mm outer diameter, iron core) [37]. iTBS consisted of bursts of 3 pulses at 50 Hz, repeated at 5 Hz for a 2 s period (10 bursts), followed by an 8 s gap, repeated for 20 cycles (total 600 pulses, 192 s). Stimulation parameters were controlled by a waveform generator using custom monophasic waveforms (400 µs rise time; Agilent Benchlink Waveform Builder), connected to a bipolar voltage programmable power supply at maximum power output (100 V) (KEPCO BOP 100-4M, TMG test equipment). Mice were habituated to the room for 1 h prior to delivering a sham stimulation or iTBS, and the intervention was delivered once per day, at the same time, for the specified duration (1 day, 2 or 4 weeks). For the ~ 3 min required, mice were gently restrained using plastic body-contour shape restraint cones (0.5 mm thick; Able Scientific). The coil was manually held over the midline of the head with the back of the coil positioned in line with the front of the ears (~ Bregma − 3.0) (Fig. S1). For sham stimulation, the procedure was the same, but no current was passed through the coil.

### Tissue preparation and immunohistochemistry

Mice were terminally anesthetised with an intraperitoneal injection of sodium pentobarbitone (150 mg/kg body weight) and transcardially perfused with 4% (w/v) paraformaldehyde (PFA; Sigma) in phosphate buffered saline (PBS; pH 7.4). Brains were cut into 2 mm-thick coronal slices using a brain matrix (Kent Scientific) before being post-fixed in 4% PFA in PBS at 21 °C for 1.5–2 h. Tissue was cryoprotected overnight in 20% (w/v) sucrose (Sigma) in PBS at 4 °C, then embedded in OCT (optimal cutting temperature matrix; Thermo Fisher Scientific) and snap frozen in liquid nitrogen for storage at − 80 °C.

Coronal brain cryosections (30 µm) containing M1 (~ Bregma + 0.5) or the secondary visual cortex (V2) (~ Bregma − 2.5) were collected and processed as floating sections [38]. Sections were pre-incubated in blocking solution [10% (v/v) fetal calf serum, 0.1% Triton X-100 in PBS, pH 7.4] for 1 h at ~ 21 °C on an orbital shaker. Primary and secondary antibodies were diluted in blocking solution and applied to cryosections overnight at 4 °C on an orbital shaker, unless staining involved the use of mouse anti-CASPR (Clone K65/35; 1:200, NeuroMab 75-001) and rabbit anti-NaV1.6 (1:300; Alamone labs), in which case sections were incubated with primary antibodies for 48 h at 4 °C. Primary antibodies included goat anti-PDGFRα (1:100, R&D Systems AF1062), rabbit anti-OLIG2 (1:400, Millipore AB9610), rabbit anti-ASPA (1:200, Abcam ab97454), rat anti-GFP (1:2000, Nacalai Tesque 04404-26), rabbit anti-c-Fos (1:5000, Abcam ab190289), mouse anti-parvalbumin (1:1000, Millipore MAB1572), and mouse anti-neun (1:300, Millipore MA377). Secondary antibodies, conjugated to AlexaFluor-488, -568 or -647 (Invitrogen) included donkey anti-goat (1:1000), donkey anti-rabbit (1:1000), donkey anti-mouse (1:1000) and donkey anti-rat (1:500). Nuclei were labelled using Hoechst 33342 (1:1000, Invitrogen). After washing in PBS, floating sections were mounted onto glass slides (Superfrost) and coverslipped with fluorescent mounting medium (Dakomount, Agilent Technologies).

### Black gold II myelin staining

Black gold II myelin staining was performed using the Biosensis Black gold II RTD kit (Sapphire Biosciences, TR-100-BG) according to the manufacturer’s instructions. Briefly, 30 µm coronal brain cryosections from ~ Bregma + 0.5 were mounted on slides and allowed to dry before being hydrated in MilliQ water for 2 min. Slides were incubated with preheated black gold II (diluted 1:10 in MilliQ water) in the dark at 65 °C for 25 min, rinsed in MilliQ water for 2 min, and incubated with sodium thiosulfate (diluted 1:10) for 3 min. Slides were washed in MilliQ water (3 × 5 min), dehydrated using a series of graded alcohol steps, cleared with xylene (Sigma, 214736) for ≥ 2 min, and mounted with DPX (Sigma, 06522).

### Confocal microscopy and cell quantification

Confocal images were obtained using an UltraView Nikon Ti Microscope with Volocity software (Perkin Elmer, Massachusetts, United States). To quantify cell number, low magnification (20×) images were taken spanning M1, V2, or the corpus callosum (CC) underlying M1. The z stack images (3 µm z-spacing) were collected using standard excitation and emission filters for DAPI, FITC (AlexaFluor-488), TRITC (AlexaFluor-568), and CY5 (AlexaFluor-647) and stitched together to make a composite image of the defined region of interest. mGFP^+^ myelinating OLs were defined using a combination of immunohistochemical and morphological criteria, as previously described [29, 39–42]. Cells were morphologically classified as myelinating OLs if they lacked PDGFRα, had a mGFP^+^ soma, and thin, straight segments of mGFP^+^ labelling (myelin internodes). To quantify internode number per mGFP^+^ OL and to measure the length of individual mGFP^+^ internodes, each mGFP^+^ OL in M1 or V2 was positioned in the centre of the field of view and a 40× image collected with 0.5 µm z-spacing. To quantify mGFP^+^ internode length in the CC, we defined a region that encompassed a mGFP^+^ cell body and its surrounding internodes and imaged the region at a high magnification (60× objective) with 0.5 µm z-spacing and stitched the images together to form a single image for analysis. Note that prior to image collection, researchers were trained to morphologically classify mGFP^+^ myelinating OLs using high magnification archival images of PDGFRα^+^ mGFP^+^ OPC, PDGFRα-neg mGFP^+^ premyelinating OL and PDGFRα-neg mGFP^+^ myelinating OLs. These images were collected from the M1 and CC of n = 4 P67 + 42 *Pdgfra-CreERT2:: Tau-mGFP* mice following immunohistochemistry to detect mGFP, PDGFRα and ASPA. ASPA labelling was removed from the training images, however, 99.2% of PDGFRα-neg mGFP^+^ myelinating OLs were ASPA^+^ (125/126 cells). To quantify black gold II myelin labelling, we collected low magnification images (2.5× objective) using a light microscope with Zeiss software.

Cell counts or internode number and length measurements were performed manually in ImageJ (NIH) or Photoshop CS6 (Adobe, San Jose, USA). The researcher performing the measurements was blind to the experimental group and treatment condition. The accuracy of quantification was verified across a subset of images by a second researcher, who was also blind to the experimental group and treatment conditions. Cell counts were carried out according to predetermined criteria. YFP^+^ or mGFP^+^ cells were only quantified when they contained a Hoechst 33342^+^ nucleus. YFP^+^ or mGFP^+^ cells were classified as PDGFRα^+^ OPCs or ASPA^+^ OLs when PDGFRα or ASPA fluorescent labelling was within the bounds of the YFP^+^/mGFP^+^ cell borders. YFP^+^ or mGFP^+^ cells were classified as OLIG2^+^ when OLIG2 labelling overlapped with the Hoechst 33342^+^ nucleus within the cell. For mGFP^+^ new OLs, the length of an individual M1, V2 or CC mGFP^+^ internode was only measured when the internode was intact i.e. the tips of the mGFP^+^ internode co-labelled with CASPR [39, 42, 43] (Fig. S2). Internodes were traced throughout each 0.5 µm z-plane to identify the complete internode prior to measuring the distance between the two mGFP^+^ CASPR^+^ ends. Within M1 and V2, the low density of mGFP^+^ new OLs allowed internodes to be attributed to an individual OL, so that the number of internodes per OL and the average length of the internodes supported by an individual OL could be quantified and reported. The high density of mGFP^+^ new OLs in the remyelinating CC did not prevent individual mGFP^+^ internodes from being identified, but they could not always be accurately assigned to a specific mGFP^+^ OL soma. Therefore, the length of individual CASPR-flanked mGFP^+^ new internodes was measured, and they were treated as a population of internodes rather than being analysed per mGFP^+^ CC OL. For mGFP^+^ surviving OLs, we identified and traced the mGFP^+^ soma through adjacent z-planes (z = 0.5 µm steps). If no discernible process could be seen extending from the soma to an internode, the surviving OL was classified as an OL without internodes; if we could discern at least one process connecting to a thin, straight stretch of mGFP^+^ labelling (a presumptive internode), then it was defined as a surviving OL that was myelinating (Fig. S3).

The number of mice in each group or the number of OLs or internodes analysed (n) is stated in each figure legend. When quantifying cell density from maximum projection images, the total number of cells within the defined region was divided by the x–y area and expressed as cells per mm^2^ (not corrected for z-depth, but tissue sections were 30 µm). We calculated the proportion (%) of YFP^+^ or mGFP^+^ cells that were OLs using the following formulae: (the number of YFP^+^ PDGFRα-neg OLIG2^+^ OLs within the defined region of interest)/(the total number of YFP^+^ cells in the region of interest) × 100. To quantify the proportion (%) of mGFP^+^ cells that were PDGFRα^+^ OLIG2^+^ OPCs or had matured into PDGFRα-neg OLIG2^+^ premyelinating or myelinating OLs, we applied the following formulae: (the number of mGFP^+^ cells of each subtype in the region of interest)/(the number of mGFP^+^ cells in the region of interest) × 100. To quantify the proportion (%) of mGFP^+^ surviving OLs with or without myelin, that were OLIG2^+^ or OLIG2-neg, the number of cells in each category was divided by the total number of mGFP^+^ cells × 100. To approximate the length of myelin sheath synthesized by individual mGFP^+^ OLs (µm), average internode length for the mGFP^+^ OL was multiplied by the number of internodes elaborated by that mGFP^+^ OL.

### Transmission electron microscopy

Mice were terminally anesthetised with an i.p. injection of sodium pentobarbitone (150 mg/kg body weight) and transcardially perfused with Karnovsky's fixative (2.5% glutaraldehyde, 2% PFA, 0.25 mM CaCl_2_, 0.5 mM MgCl_2_ in 0.15 M sodium‐cacodylate buffer). Brains were sliced into 1 mm thick coronal slices using a brain matrix (Kent Scientific) and post-fixed in Karnovsky's fixative at ~ 21 °C for 2 h, before being stored in 0.15 M sodium‐cacodylate buffer overnight at 4 °C. M1 (~ Bregma + 1.0 to 0) and the CC (~ Bregma 0 to − 1) were dissected and immersed in 2% osmium tetroxide and then 2.5% potassium ferricyanide in 0.15 M sodium‐cacodylate buffer in the dark for 1.5 h each at 4 °C and 21 °C, respectively. Tissues were washed thrice in Milli‐Q water, stained with 1% uranyl acetate overnight at 4 °C, and stored at 50 °C for 1 h. Tissues were washed in Milli‐Q water (2 × 15 min) and dehydrated [30% ethanol for 10 min at 21 °C; 50% ethanol for 10 min at 21 °C; 70% ethanol for 10 min at 21 °C; 80% ethanol for 10 min on ice; 90% ethanol for 10 min on ice; 95% ethanol for 5 min on ice; 100% ethanol (2 × 5 min on ice and 2 × 5 min at 21 °C); and 100% propylene oxide (2 × 10 min at 21 °C)]. Tissue was embedded in Epon812 resin and cut into ultrathin 70 nm sections using a Leica Ultra-cut UCT7. Imaging was performed at 80 kV on a Hitachi HT7700 transmission electron microscope.

Image analysis was carried out using ImageJ (NIH) by an experimenter blind to the treatment group. Axons were identified by the pattern of transected microtubules. The proportion of myelinated axons was calculated after classifying ≥ 100 axons spanning a minimum of 3 images (8–10,000× magnification) per mouse. The g-ratio was calculated from manual measurement of the myelinated axons [axon diameter/(axon + myelin diameter)].

### Statistical analyses

All statistical analyses were performed using Prism 8 (GraphPad Software). When data were normally distributed, two groups were compared using an unpaired two-tailed t-test, and when data were not normally distributed, they were analysed using a Mann–Whitney U test, as specified in the figure legend. For internode length, internode number or myelin load per OL data, the data were nested within mice and analysed using a nested t-test. Internode length, g-ratio and axon diameter cumulative distributions were analysed using Kolmogorov–Smirnov (KS) tests. When comparing cell counts across treatment conditions and/or myelination status, but also between anatomical regions of the same mice, data were analysed using mixed two-way or three-way ANOVAs, with region as the repeated measures factor (Greenhouse–Geisser corrected if sphericity was violated), and treatment condition and/or myelination status as the between-subjects factor(s). Follow-up tests for simple effects of treatment within each brain region were Bonferroni corrected for multiple comparisons. To determine whether the mean differs from 0, data for each group were analysed separately using a one sample t-test or Wilcoxon signed-rank test to compare the group mean or median, respectively, to 0. Multiple comparisons were corrected (with a false discovery rate set at 5%) using the Benjamini–Hochberg method. Statistical significance was defined as p < 0.05, corrected for multiple testing. Further statistical information is provided in each figure legend including, for example, ANOVA main effects.

### Data availability

The image files and data described in this manuscript can be obtained from the corresponding author by reasonable request.

## Results

### When delivered during CPZ feeding, iTBS does not alter new OL number in the cortex or CC

**Fig. 1:**
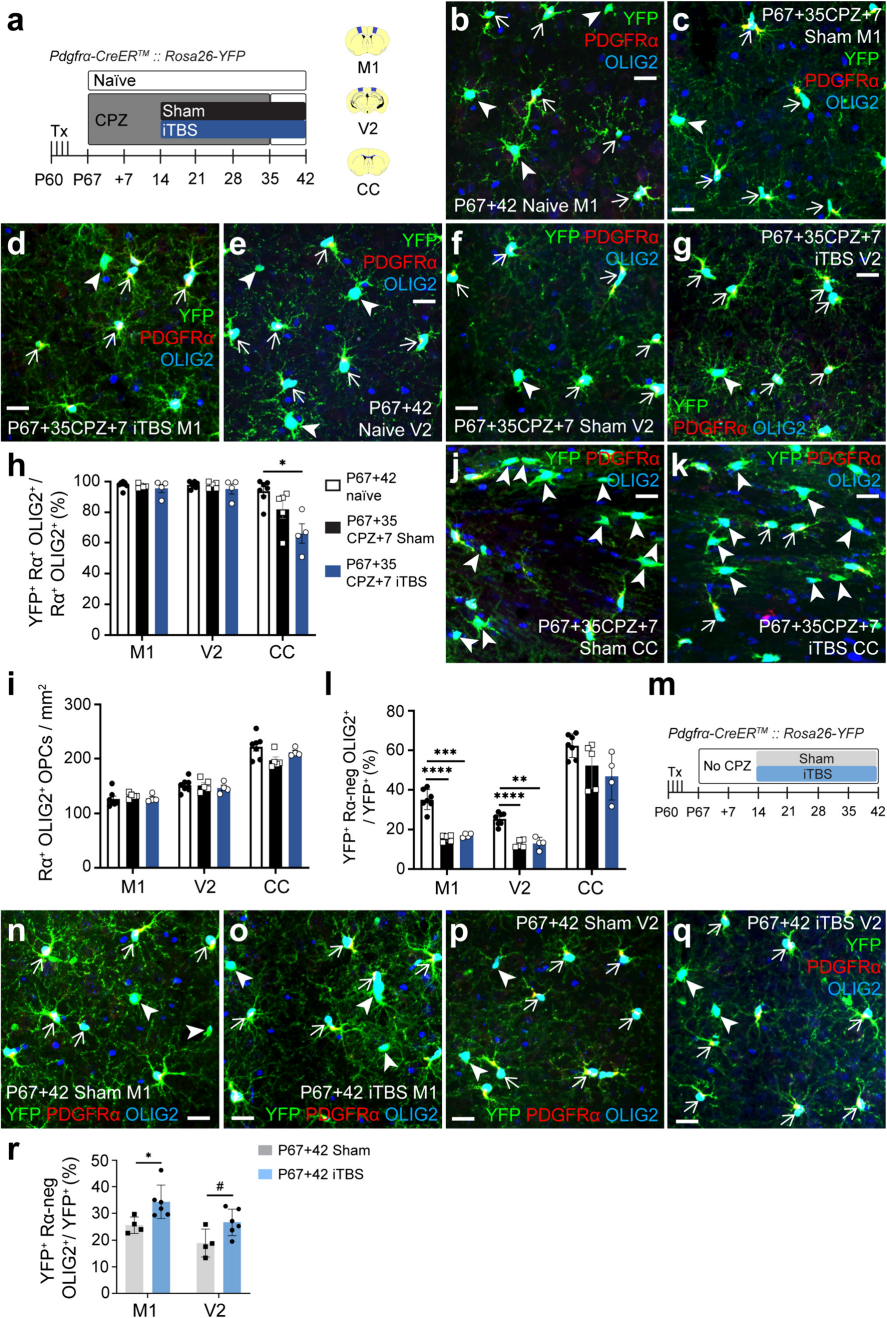
iTBS during demyelination does not increase OL addition to the cortex or corpus callosum. **a** Experimental schematic showing the timeline over which *Pdgfrα-CreER™:: Rosa26-YFP* mice received 4 consecutive days of Tx, up to 35 days of CPZ and up to 28 days of LI-rTMS (sham-stimulation or iTBS). This experiment included three treatment groups: P67+42 naïve, P67+35CPZ+7 sham, and P67+35CPZ+7 iTBS. The brain regions analysed are shown in blue in the coronal brain section schematics: M1 (~ Bregma + 0.5), V2 (~ Bregma − 2.5) and the CC (~ Bregma + 0.5). **b**–**g** Confocal images of YFP (green), PDGFRα (red) and OLIG2 (blue) immunohistochemistry in M1 (**b**–**d**) and V2 (**e**–**g**) of P67 + 42 naïve (**b**, **e**), P67+35CPZ+7 sham (**c**, **f**) and P67+35CPZ+7 iTBS (**d**, **g**) mice. YFP^+^ PDGFRα^+^ OPCs are denoted by arrows. YFP^+^ PDGFRα-neg OLs are denoted by arrowheads. **h** The proportion (%) of PDGFRα^+^ OLIG2^+^ OPCs that expressed YFP in the M1, V2 and CC of P67 + 42 naïve (white, n = 7), P67+35CPZ+7 sham (black, n = 5) or P67+35CPZ+7 iTBS (blue, n = 4) mice. Repeated measures two-way ANOVA with Geisser–Greenhouse correction: treatment F (2, 13) = 5.30, p = 0.02; region F (1.03, 13.35) = 48.37, p < 0.0001; interaction F (4, 26) = 9.71, p < 0.0001. **i** Density of PDGFRα^+^ OLIG2^+^ OPCs in M1, V2 and the CC of P67+42 naïve (white, n = 7), P67+35CPZ+7 sham (black, n = 5) or P67+35CPZ+7 iTBS (blue, n = 4) mice. Repeated measures two-way ANOVA with Geisser–Greenhouse correction: treatment F (2, 13) = 0.92, p = 0.42; region F (1.94, 25.26) = 188.5, p < 0.0001; interaction F (4, 26) = 2.82, p = 0.045. **j**–**k** Confocal images of YFP (green), PDGFRα (red) and OLIG2 (blue) immunohistochemistry in the CC of P67 + 35CPZ + 7 sham (**j**) and P67+35CPZ+7 iTBS (**k**) mice. **l** The proportion (%) of YFP^+^ cells that are newly differentiated OLs (PDGFRα-neg OLIG2^+^) in M1, V2 and CC of P67+42 naïve (white, n = 7), P67+35CPZ+7 sham (black, n = 5) or P67+35CPZ+7 iTBS (blue, n = 4) mice. Repeated measures two-way ANOVA with Geisser–Greenhouse correction: treatment F (2, 13) = 22.63, p < 0.0001; region F (1.23, 15.98) = 216.1, p < 0.0001; interaction F (4, 26) = 1.53, p = 0.222. **m** Experimental schematic showing the time course over which *Pdgfrα-CreER™:: Rosa26-YFP* mice received Tx and 28 days of LI-rTMS (sham-stimulation or iTBS). The experiment had two groups: P67+42 sham and P67+42 iTBS. **n**–**q** Confocal images of YFP (green), PDGFRα (red) and OLIG2 (blue) immunohistochemistry in M1 (**n**, **o**) or V2 (**p**, **q**) of P67 + 42 sham (**n**, **p**) and P67+42 iTBS (**o**, **q**) mice. **r** The proportion (%) of YFP^+^ cells that are PDGFRα-neg OLIG2^+^ OLs in the M1 and V2 cortices of P67 + 42 sham (n = 4) and P67+42 iTBS (n = 6) mice. Repeated measures two-way ANOVA: treatment F (1, 8) = 7.44, p = 0.03; region F (1, 8) = 26.09, p = 0.0009; interaction F (1, 8) = 0.1305, p = 0.727. Data are presented as mean ± SD. Bonferroni post-test: *p < 0.05, ****p < 0.0001, ^#^p = 0.067. Scale bars represent 20 µm

In the CPZ model of demyelination, OPCs proliferate and differentiate, but many of the new OLs die unless CPZ is withdrawn [8]. We aimed to determine whether iTBS delivery during CPZ feeding and in the first week following CPZ withdrawal, can increase the number of new OLs added to the cortex or CC. Tx was administered to P60 *Pdgfrα-CreER™:: Rosa26-YFP* transgenic mice to fluorescently label OPCs throughout the CNS (Fig. 1a). From P67, mice were maintained on a control diet (treatment naïve mice, P67+42) or transferred to a CPZ diet for 35 days to induce demyelination before being returned to control chow for a further 7 days to enable remyelination (P67+35CPZ+7). On day 14 of CPZ feeding, mice commenced sham stimulation or iTBS, which was delivered daily for 28 consecutive days i.e. for the remainder of the 42-day time-course (Fig. 1a). Coronal brain cryosections were immunolabelled to detect YFP, PDGFRα (OPCs) and OLIG2 (all cells of the OL lineage) in M1, V2 and the CC (Fig. 1b–g). These regions were selected for analysis as they are demyelinated by CPZ-feeding [8, 44–46] and cells within these regions respond to LI-rTMS in healthy mice [29, 42]. We found that > 95% of PDGFRα^+^ parenchymal M1 and V2 OPCs were YFP-labelled in P67+42 naïve and P67+35CPZ+7 sham or iTBS *Pdgfrα-CreER™:: Rosa26-YFP* mice (Fig. 1h). Total M1 or V2 OPC density was also equivalent in P67+42 naïve and P67+35CPZ+7 sham or iTBS mice (Fig. 1i). While > 95% of PDGFRα^+^ OPCs were similarly YFP-labelled in the CC of P67+42 naïve mice, reflecting the initial recombination efficiency (Fig. 1h), fewer OPCs were YFP^+^ in the CC of P67+35CPZ+7 sham or iTBS mice (Fig. 1h, j, k). This likely reflects the increased generation of YFP-neg PDGFRα^+^ OPCs from subventricular zone neural stem cells following callosal demyelination [40, 47]. As the same proportion of callosal OPCs was YFP-labelled in P67+35CPZ+7 sham and iTBS *Pdgfrα-CreER™:: Rosa26-YFP* mice (Fig. 1h), iTBS does not appear to enhance OPC generation from neural stem cells. Furthermore, as total PDGFRα^+^ callosal OPC density was equivalent in naïve and P67+35CPZ+7 sham or iTBS mice (Fig. 1i), iTBS does not affect OPC density in this region.

YFP^+^ OPCs produced new YFP^+^ OLs (OLIG2^+^ PDGFRα-neg cells) in M1 (Fig. 1b–d), V2 (Fig. 1e–g) and the CC (Fig. 1j, k) of P67+42 naïve and P67+35CPZ+7 sham or iTBS mice. A higher proportion of the YFP^+^ cells survived as new OLs in the M1 or V2 cortex of P67+42 naïve mice relative to P67+35CPZ+7 sham or iTBS mice (Fig. 1l), indicating that the CPZ diet killed many new OLs. By contrast, the proportion of YFP^+^ cells that were new callosal OLs was equivalent in P67 + 42 naïve and P67+35CPZ+7 sham or iTBS treated *Pdgfrα-CreER™:: Rosa26-YFP* mice (Fig. 1l), consistent with the rapid replacement of callosal OLs after CPZ withdrawal. Importantly, the proportion of YFP^+^ OPCs that differentiated and/or survived as new M1, V2 or CC OLs was not altered by iTBS (Fig. 1l), suggesting that iTBS does not alter the generation or survival of new OLs during demyelination.

As Tx delivery labelled OPCs at P60, 3 weeks prior to sham or iTBS delivery (Fig. 1a), many of the new YFP^+^ OLs differentiated or died without being influenced by the intervention. To evaluate the impact of this time-course on our study conclusions, we delivered Tx to healthy P60 *Pdgfrα-CreER™:: Rosa26-YFP* mice, and waited 3 weeks before commencing 4 weeks of sham stimulation or iTBS (from P67+14 to P67+42) (Fig. 1m). Mice that received iTBS had ~ 34% more YFP^+^ OLs in M1 and ~ 41% more in V2 than sham-stimulated mice (Fig. 1n–r). These data are consistent with our previous report that iTBS promotes new OL survival in the healthy mouse cortex [29]. Commencing iTBS 3 weeks after lineage tracing (instead of our previous 1 week; [29]) reduced the apparent effect size, but did not prevent the detection of iTBS-enhanced OL survival. This experiment provides greater confidence in our conclusion that iTBS promotes new OL survival in the healthy CNS but not during CPZ-feeding.

### iTBS increases the number of mGFP^+^ internodes elaborated by new M1 OLs

**Fig. 2:**
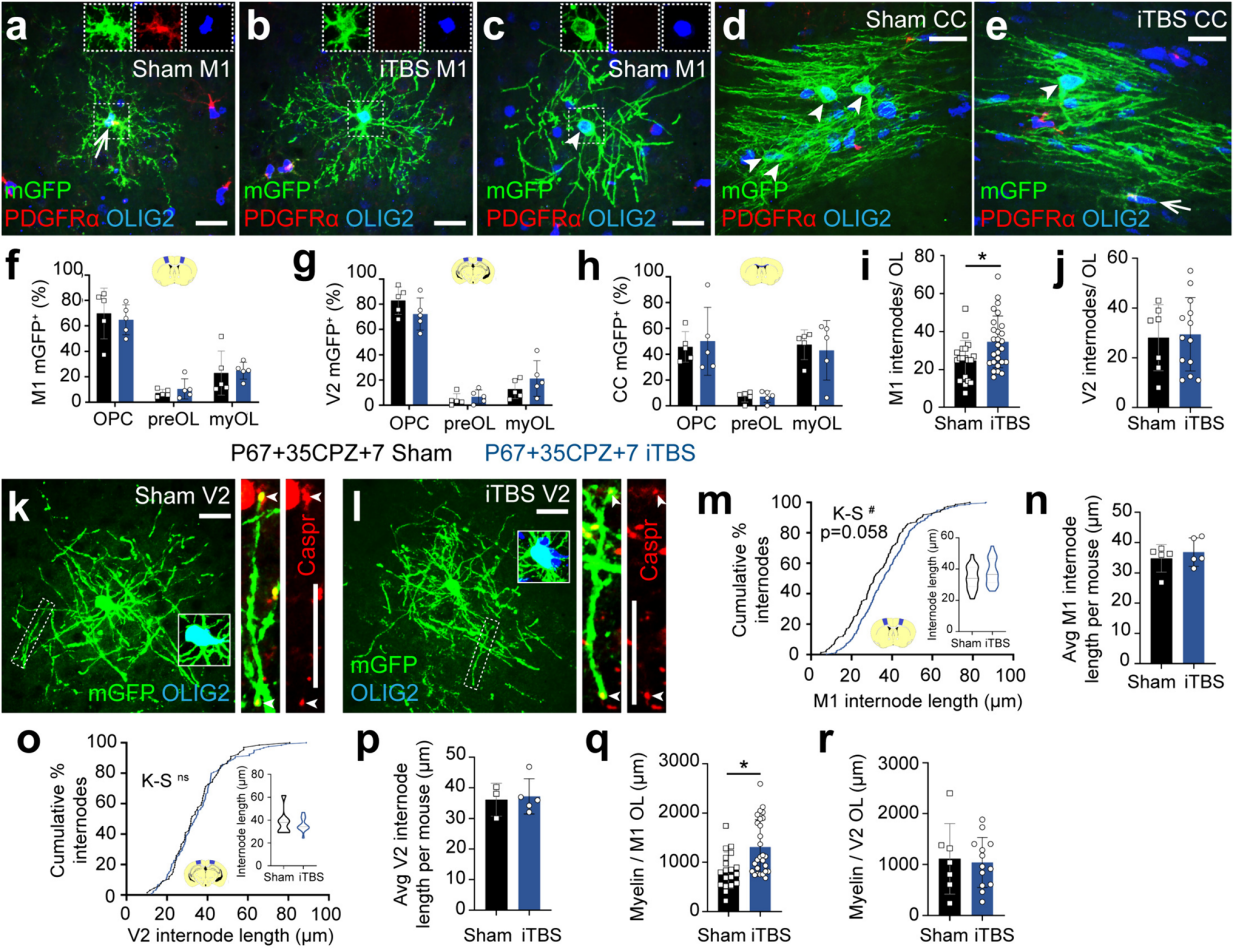
iTBS increases the number of internodes elaborated by new OLs in M1 of CPZ-fed mice. **a**–**e** Confocal images of mGFP (green), PDGFRα (red) and OLIG2 (blue) immunohistochemistry in M1 (**a**–**c**) and the CC (**d**, **e**) of *Pdgfrα-CreER*^*T2*^*:: Tau-mGFP* P67+35CPZ+7 sham (**a**, **c**, **d**) and iTBS (**b**, **e**) mice. Arrows denote OPCs. Arrowheads denote myelinating OLs. **f**–**h** The proportion (%) of mGFP^+^ cells that are OPCs, premyelinating OLs or myelinating OLs in M1 (**f**), V2 (**g**) and the CC (**h**) of P67+35CPZ+7 sham (black, n = 5) and P67+35CPZ+7 iTBS (blue, n = 5) mice. 95% confidence intervals overlap for P67+35CPZ+7 sham and iTBS mice, indicating no change in cell-type distribution. **i**–**j** Quantification of the number of internodes produced by individual mGFP^+^ myelinating OLs in M1 (**i**) or V2 (**j**) of P67+35CPZ+7 sham or iTBS mice [M1: n = 19 OLs from n = 5 sham mice v n = 27 OLs from n = 5 iTBS mice in nested t-test, p = 0.025; V2: n = 7 OLs from n = 3 sham mice v n = 13 OLs from n = 5 iTBS mice in nested t-test, p = 0.63]. **k**–**l** Confocal images of mGFP (green), OLIG2 (blue; main panel insets) and CASPR (red; righthand outset) in V2 of P67+35CPZ+7 sham (**k**) or iTBS (**l**) mice. The outset is a higher magnification of the region denoted by the dashed box, showing a single mGFP^+^ internode flanked by CASPR^+^ (red) paranodes (arrowheads). **m** Cumulative distribution plot of M1 mGFP^+^ internode length for P67+35CPZ+7 sham (black) or iTBS (blue) mice [n = 148 sham and n = 248 iTBS mGFP^+^ internodes, Kolmogorov–Smirnov (K–S) test D = 0.14, p = 0.058]. Inset violin plot for average internode length per mGFP^+^ myelinating OL in M1 of P67+35CPZ+7 sham or iTBS mice [n = 19 mGFP^+^ OLs from n = 5 sham mice vs n = 27 mGFP^+^ OLs from n = 5 iTBS mice, nested t-test, p = 0.3]. **n** Average M1 mGFP^+^ internode length per mouse for P67+35CPZ+7 sham (n = 5) and iTBS (n = 5) mice [unpaired t-test, p = 0.5]. **o** Cumulative distribution plot of V2 mGFP^+^ internode length for P67+35CPZ+7 sham (black) or iTBS (blue) mice [n = 66 sham and n = 119 iTBS mGFP^+^ internodes, K–S test D = 0.08, p = 0.95]. Inset violin plot of average internode length per mGFP^+^ myelinating OL in V2 of P67+35CPZ+7 sham or P67+35CPZ+7 iTBS mice [n = 7 mGFP^+^ OLs from n = 3 sham mice vs n = 13 mGFP^+^ OLs from n = 5 iTBS mice, nested t-test, p = 0.55]. **p** Average V2 mGFP^+^ internode length per mouse for P67+35CPZ+7 sham (n = 3) and iTBS (n = 5) mice [unpaired t-test, p = 0.79]. **q** Estimate of myelin load per mGFP^+^ myelinating OL in M1 of P67+35CPZ+7 sham or iTBS mice (avg. internode length per OL x avg. number of internodes per OL) [n = 19 OLs from n = 5 sham mice v n = 27 OLs from n = 5 iTBS mice; nested t-test, p = 0.016). **r** Estimate of myelin load per mGFP^+^ OL in V2 of P67+35CPZ+7 sham or iTBS mice [n = 7 OLs from n = 3 sham mice v n = 13 OLs from n = 5 iTBS mice; nested t-test, p = 0.86]. Violin plots show the median (solid line) and interquartile range (dashed lines). Data are presented as mean ± SD. *p < 0.05, ^#^p = 0.058. Scale bars represent 20 µm

If iTBS alters the number or length of myelin internodes produced by new OLs [29], it could promote remyelination without increasing the number of new OLs added to the brain. To examine this possibility, we delivered Tx to P60 *Pdgfrα-CreER*^*T2*^*:: Tau-mGFP* mice to label OPCs and reveal the full morphology of the mGFP^+^ premyelinating and myelinating OLs they produce. Mice were transferred to a CPZ diet from P67-P102 and returned to a control diet for 1 week. Mice received daily sham or iTBS from P67+14 (as per Fig. 1a). Coronal brain sections from P67+35CPZ+7 *Pdgfrα-CreER*^*T2*^*:: Tau-mGFP* mice, that included M1, V2 or CC, were immunolabeled to detect mGFP, PDGFRα and OLIG2 (Fig. 2a–e). In all regions examined, mGFP^+^ OPCs (Fig. 2a) had differentiated to produce new mGFP^+^ premyelinating (Fig. 2b) and myelinating OLs (Fig. 2c–e). Premyelinating OLs were highly branched, PDGFRα-negative cells (Fig. 2b) that lacked the straight sections of mGFP^+^ labelling that denoted the myelin internodes of mature OLs (Fig. 2c–e and Fig. S4).

iTBS did not alter the proportion of mGFP^+^ cells that were OPCs, premyelinating or myelinating OLs in M1, V2 or the CC (Fig. 2f–h). However, a more detailed analysis of the mGFP^+^ myelinating OLs revealed that iTBS increased the average number of mGFP^+^ internodes elaborated by new M1 myelinating OLs (~ 25 internodes/OL in sham v ~ 35 internodes/OL in iTBS; Fig. 2i). iTBS did not alter the average number of internodes elaborated by mGFP^+^ myelinating OL in V2 (Fig. 2j). The end of mGFP^+^ internodes can be identified by colabelling with the paranodal protein contactin-associated protein (CASPR) (Fig. 2k, l; Fig. S2). We identified a trend towards new OLs elaborating longer mGFP^+^ internodes in M1 following iTBS (K–S test, p = 0.058, n = 148 sham and 248 iTBS internodes; Fig. 2m). However, average internode length per mouse was comparable in sham-stimulated and iTBS mice (Fig. 2n). mGFP^+^ internode length distribution and average internode length per mouse were equivalent in the V2 of sham-stimulated and iTBS mice (Fig. 2o, p). To obtain an approximation of myelin load per OL, we multiplied the average number of internodes produced per OL with the average internode length. For sham-stimulated mice, we estimate that mGFP^+^ myelinating M1 OLs support ~ 840 µm of myelin, but this increased to ~ 1311 µm in iTBS mice (Fig. 2q). By contrast, in V2 the mGFP^+^ OL myelin load was equivalent in sham and iTBS mice (Fig. 2r). These data suggest that delivering iTBS during demyelination and early CPZ withdrawal can enhance the remyelinating capacity of new myelinating OLs within M1, largely by increasing the average number of internodes elaborated per cell.

### iTBS delivery after CPZ withdrawal does not alter new OL number in the cortex or CC

**Fig. 3:**
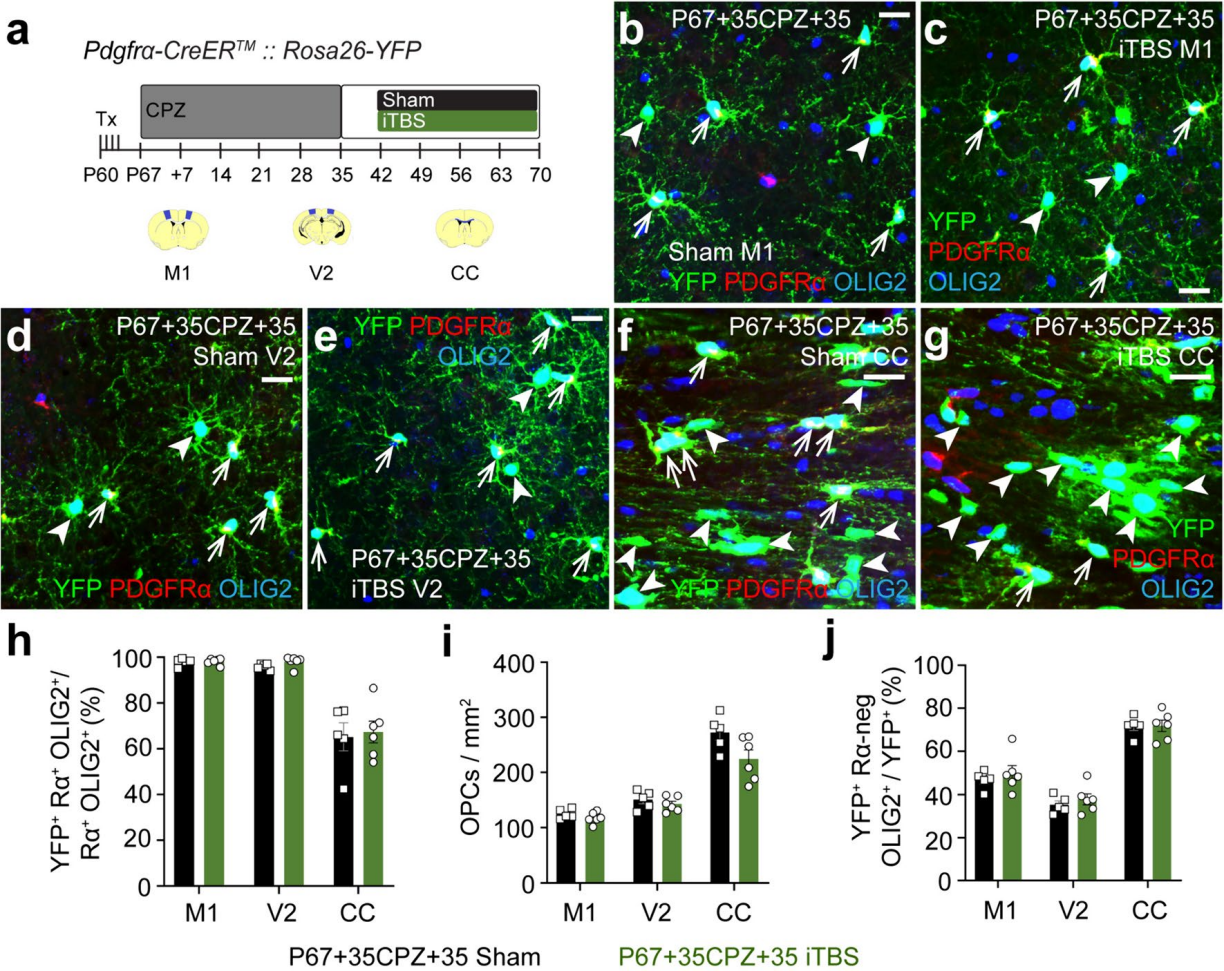
iTBS does not increase new OL number in the cortex or CC after CPZ withdrawal. **a** Experimental schematic showing the timeline over which *Pdgfrα-CreER™:: Rosa26-YFP* mice received 4 consecutive days of Tx, 35 days of CPZ and up to 28 days of LI-rTMS (sham-stimulation or iTBS). This experiment included two treatment groups: P67+35CPZ+35 sham and iTBS. The brain regions analysed are shown in blue in the coronal brain section schematics: M1 (~ Bregma + 0.5), V2 (~ Bregma − 2.5) and the CC (~ Bregma + 0.5). **b**–**g** Confocal images of YFP (green), PDGFRα (red) and OLIG2 (blue) immunohistochemistry in M1 (**b**, **c**), V2 (**d**, **e**) and CC (**f**, **g**) of P67+35CPZ+35 sham (**b**, **d**, **f**) or iTBS (**c**, **e**, **g**) mice. Arrows indicate YFP^+^ OPCs. Arrowheads indicate new YFP^+^ OLs. **h** The proportion (%) of PDGFRα^+^ OLIG2^+^ OPCs that are YFP-labelled in M1, V2 and the CC of P67+35CPZ+35 sham (n = 5) and iTBS (n = 6) mice. Repeated measures two-way ANOVA with Geisser–Greenhouse correction: treatment F (1, 9) = 0.26, p = 0.62; region F (1.01, 9.11) = 75.14, p < 0.0001; interaction F (2, 18) = 0.077, p = 0.9. **i.** The density of PDGFRα^+^ OLIG2^+^ OPCs in M1, V2 and the CC of P67+35CPZ+35 sham (n = 5) and iTBS (n = 6) mice. Repeated measures two-way ANOVA with Geisser–Greenhouse correction: treatment F (1, 9) = 5.9, p = 0.04; region F (1.22, 10.99) = 96.8, p < 0.0001; interaction F (2, 18) = 2.86, p = 0.08. **j** The (%) of YFP^+^ cells that are new OLs (YFP^+^ PDGFRα-neg OLIG2^+^) in M1, V2 and the CC of P67+35CPZ+35 sham (n = 5) and iTBS (n = 6) mice. Repeated measures two-way ANOVA with Geisser–Greenhouse correction: treatment F (1, 9) = 0.76, p = 0.41; region F (1.28, 11.48) = 96.96, p < 0.0001; interaction F (2, 18) = 0.19, p = 0.83. Data are presented as mean ± SD. Scale bars represent 20 µm

iTBS did not increase new OL number when delivered during CPZ feeding (Figs. 1, 2). To explore the ability of iTBS to increase new OL number if delivered after CPZ withdrawal, *Pdgfrα-CreER™:: Rosa26-YFP* transgenic mice received Tx and were transferred onto a CPZ diet for 35 days before being returned to a control diet to allow remyelination (Fig. 3a). Mice received sham-stimulation or iTBS daily for 28 days from P67+42 (7 days after CPZ withdrawal) and tissue was collected at P67+70 for immunohistochemistry to detect YFP, PDGFRα and OLIG2 (Fig. 3a–g). ~ 98% of M1, ~ 96% of V2, and ~ 65% of CC PDGFRα^+^ OPCs were YFP-labelled in sham-stimulated P67+35CPZ+35 mice, and the labelled fraction was unchanged by iTBS (Fig. 3h). OPC density was also equivalent (Fig. 3i). YFP^+^ PDGFRα-neg OLIG2^+^ new OLs were readily detected in M1 (Fig. 3b, c), V2 (Fig. 3d, e) and CC (Fig. 3f, g) of sham and iTBS mice, and the proportion of YFP^+^ cells that were new OLs was unaltered by iTBS (M1: sham ~ 47%, iTBS ~ 50%; V2: sham ~ 35%, iTBS ~ 38%; CC: ~ 72% in sham and iTBS; Fig. 3j).

### iTBS increases the length of remyelinating internodes in M1 and the CC

**Fig. 4:**
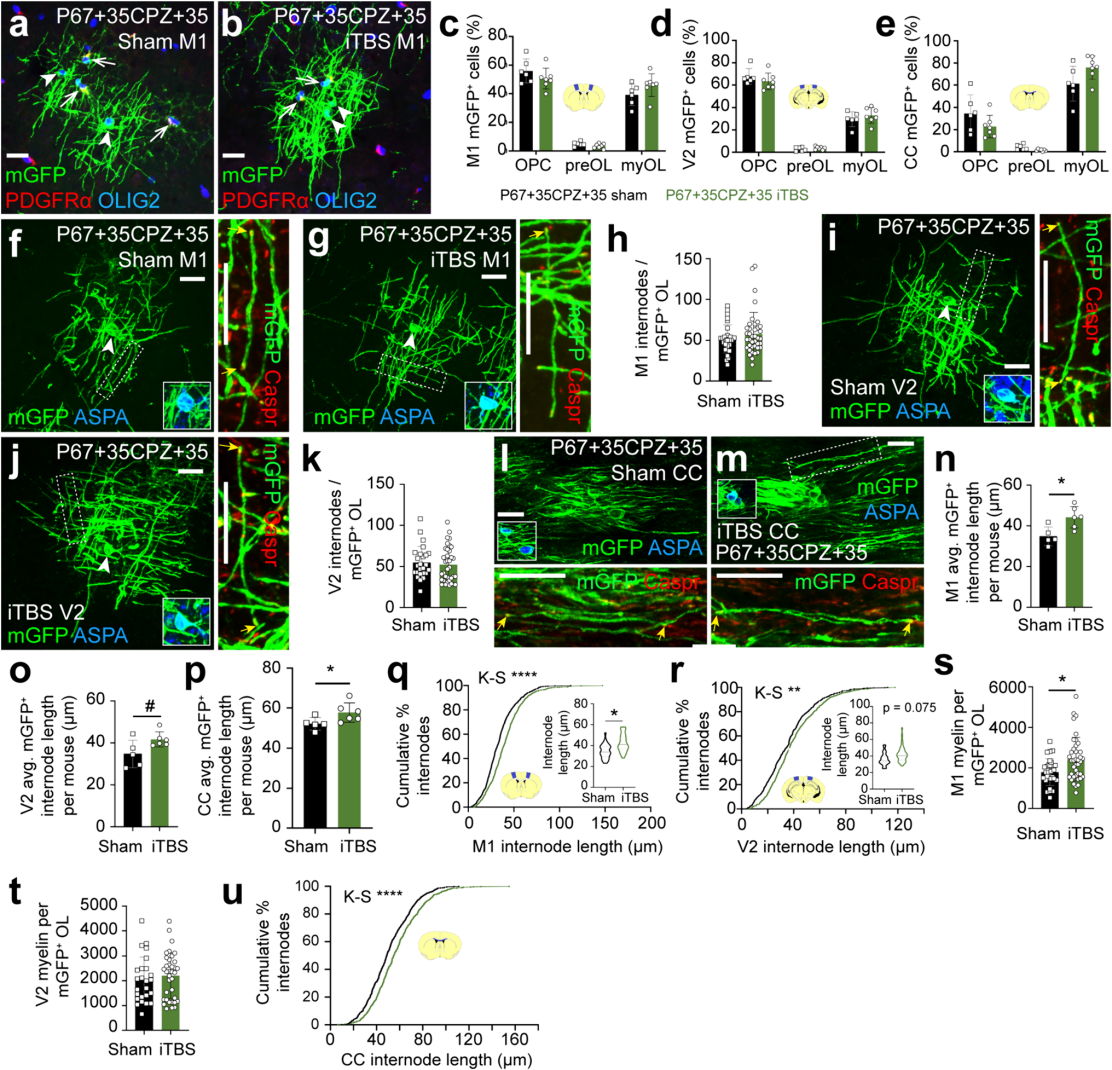
iTBS increases the length of internodes elaborated by new OLs in M1 and the CC after CPZ withdrawal. **a**, **b** Representative confocal images of mGFP (green), PDGFRα (red) and OLIG2 (blue) immunohistochemistry in M1 cortex of *Pdgfrα-CreER*^*T2*^*:: Tau-mGFP* P67+35CPZ+35 sham (**a**) and iTBS (**b**) mice. Arrows indicate mGFP^+^ OPCs. Arrowheads indicate PDGFRα-neg OLIG2^+^ mGFP^+^ myelinating OLs. **c**–**e** The proportion (%) of mGFP cells that are OPCs, premyelinating OLs and myelinating OLs per mouse in M1 (**c**), V2 (**d**) and CC (**e**) of P67 + 35CPZ + 35 sham (n = 6) or iTBS (n = 7) mice. 95% confidence intervals overlap for P67+35CPZ+35 sham and iTBS mice, indicating no change in cell-type distribution. **f**–**g** Confocal images of mGFP (green), ASPA (blue, inset showing cell body) and CASPR (red, outset showing internode) immunohistochemistry in M1 of P67+35CPZ+35 sham (**f**) and iTBS (**g**) mice. Outset shows higher magnification of dashed box with paranodes indicated by small arrows. **h.** The number of internodes elaborated by individual M1 mGFP^+^ myelinating OL in P67+35CPZ+35 sham or iTBS mice (n = 30 OLs from n = 5 sham mice vs n = 39 OLs from n = 6 iTBS mice; nested t-test, p = 0.335). **i**–**j** Confocal images of mGFP (green), ASPA (blue, inset showing cell body) and CASPR (red, outset showing internode) immunohistochemistry in V2 of P67+35CPZ+35 sham (**i**) and iTBS (**j**) mice. Outset shows higher magnification of dashed box with paranodes indicated by small arrows. **k** The number of internodes elaborated by individual V2 mGFP^+^ myelinating OL in P67+35CPZ+35 sham or iTBS mice (n = 25 OLs from n = 5 sham mice vs n = 36 OLs from n = 6 iTBS mice; nested t-test, p = 0.73). **l**–**m** Single confocal z-plane image of mGFP (green), ASPA (blue, inset showing cell body) and CASPR (red, outset showing internode) immunohistochemistry in the CC of P67+35CPZ+35 sham (**l**) and iTBS (**m**) mice. Outset shows higher magnification of adjacent z-plane (l) or dashed box (m) with paranodes indicated by small arrows. **n**–**p** Average mGFP^+^ internode length per mouse in M1 (**n**), V2 (**o**) or CC (**p**) of P67+35CPZ+35 sham (n = 5) and iTBS (n = 6) mice [M1 MWU test, p = 0.012; V2 unpaired t-test, p = 0.05; CC MWU test, p = 0.03]. **q**–**r** Cumulative distribution plot of M1 (**q**) or V2 (**r**) mGFP^+^ internode length for P67+35CPZ+35 sham (black) and iTBS (green) mice [M1: n = 538 mGFP^+^ internodes from n = 5 sham mice vs n = 713 mGFP^+^ internodes from n = 6 iTBS mice, K–S test D = 0.184, p < 0.0001; V2: n = 427 mGFP^+^ internodes from n = 5 sham mice vs n = 730 mGFP^+^ internodes from n = 6 iTBS mice, K–S test D = 0.11, p = 0.004]. Inset violin plots of average mGFP^+^ internode length for individual mGFP^+^ myelinating OLs in P67+35CPZ+35 sham and iTBS mice [M1: n = 30 mGFP^+^ OLs from n = 5 sham mice vs n = 39 mGFP^+^ OLs from n = 6 iTBS mice, nested t-test, p = 0.017; V2: n = 25 mGFP^+^ OLs from n = 5 sham mice vs n = 36 mGFP^+^ OLs from n = 6 iTBS mice, nested t-test, p = 0.075]. **s**–**t** Estimate of myelin load per mGFP^+^ OL in M1 (**s**) and V2 (**t**) of P67 + 35CPZ + 35 sham or iTBS mice (avg. internode length per OL x avg. number of internodes per OL) [M1: n = 30 OLs from n = 5 sham mice v n = 39 OLs from n = 6 iTBS mice, nested t-test, p = 0.04; V2: n = 25 OLs from n = 5 sham mice v n = 36 OLs from n = 6 iTBS mice, nested t-test, p = 0.6). **u** Cumulative distribution plot of CC mGFP^+^ internode length for P67+35CPZ+35 sham (black) and iTBS (green) mice [n = 632 mGFP^+^ internodes from n = 5 sham mice vs n = 680 mGFP^+^ internodes from n = 6 iTBS mice, K–S test D = 0.107, p < 0.0001]. Data are presented as mean ± SD. Violin plots show the median (solid line) and interquartile range (dashed lines). *p < 0.05, **p < 0.01, ****p < 0.0001, ^#^p = 0.052. Scale bars represent 20 µm

To determine whether iTBS altered new OL maturation in remyelinating mice, we delivered Tx to P60 *Pdgfrα-CreER*^*T2*^*:: Tau-mGFP* mice to fluorescently label a subset of OPCs and trace their generation of mGFP^+^ premyelinating and myelinating OLs until P67+35CPZ+35 (Fig. 4). Mice received daily sham stimulation or iTBS, commencing 7 days after CPZ withdrawal (as per Fig. 3a). We examined the morphology of individual mGFP^+^ cells to classify each as an OPC (PDGFRα^+^ OLIG2^+^), premyelinating or myelinating OL (PDGFRα-neg OLIG2^+^; Fig. 4a, b). Approximately 40% of the mGFP^+^ cells were myelinating OLs in M1 (Fig. 4c) and ~ 30% in V2 of sham-stimulated mice (Fig. 4d) - significantly less than the ~ 61% of mGFP^+^ cells that were myelinating OLs in the CC (Fig. 4e). These data are consistent with a higher rate of OL differentiation and remyelination in the CC relative to the cortex following CPZ withdrawal [8]. The proportion of mGFP^+^ cells that were premyelinating or myelinating OLs was unaffected by iTBS (Fig. 4c–e).

By performing immunohistochemistry to detect mGFP, the mature OL marker ASPA, and the paranodal marker CASPR, and collecting high magnification images of mGFP^+^ myelinating OLs in M1 (Fig. 4f–h) and V2 (Fig. 4i–k), we determined that mGFP^+^ ASPA^+^ OLs elaborate an equivalent number of internodes in P67+35CPZ+35 sham and iTBS mice [M1: ~ 52 internodes/sham OL v ~ 58 internodes/iTBS OL; V2: ~ 55 internodes/sham OL v ~ 52 internodes/iTBS OL]. The high density of mGFP^+^ OLs within the remyelinating CC made it impossible to attribute a full complement of internodes to an individual OL soma (Fig. S5). However, it was still possible to identify mGFP^+^ internodes that were intact and clearly tipped by CASPR^+^ paranodes. Therefore, we measured the length of individual mGFP^+^ internodes in M1 (Fig. 4f, g), V2 (Fig. 4i, j) and the CC (Fig. 4l, m). In sham-stimulated mice, new cortical internodes were shorter than new callosal internodes [M1 mean ~ 35 µm (Fig. 4n), V2 mean ~ 35 µm (Fig. 4o), CC mean ~ 52 µm (Fig. 4p)]. Furthermore, iTBS delivery shifted M1 (Fig. 4q) and V2 (Fig. 4r) internode length distributions toward the right, indicative of longer internodes. This corresponded to a ~ 21% increase in mean internode length per mouse in M1 (Fig. 4n, p = 0.012) and a ~ 17% increase in V2 (Fig. 4o, p = 0.052). iTBS increased the myelin load of individual mGFP^+^ M1 OLs by ~ 645 µm (Fig. 4s). By contrast, iTBS did not significantly increase the quantity of myelin produced by new mGFP^+^ OLs in V2 (Fig. 4t). Within the CC, iTBS also produced a significant right-ward shift in internode length distribution, indicative of longer internodes in the CC (Fig. 4u), which corresponded to a ~ 10% increase in average internode length per mouse (Fig. 4p). These data suggest that iTBS may facilitate remyelination by enhancing internode extension by new OLs in M1 and the CC.

### iTBS increases the activity of PV^+^ interneurons in M1 and V2

**Fig. 5:**
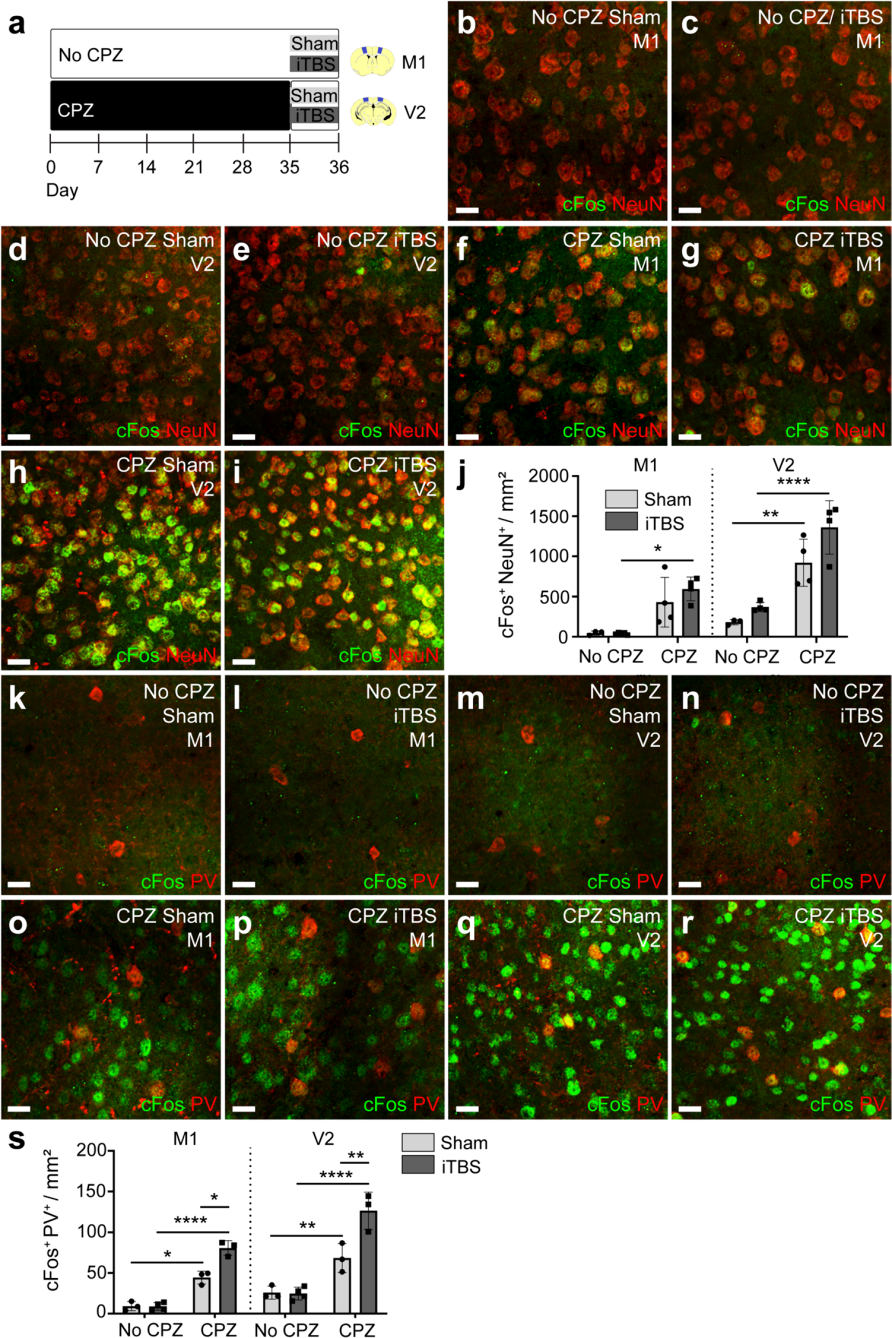
A single session of iTBS increases the density of cFos^+^ parvalbumin interneurons in the demyelinated cortex. **a** Schematic showing the timeline over which mice received up to 35 days of CPZ-feeding and one session of LI-rTMS (sham or iTBS). **b**–**i** Confocal images of cFos (green) and neun (red) immunohistochemistry in M1 (**b**, **c**, **f**, **g**) and V2 (**d**, **e**, **h**, **i**) of no CPZ sham (**b**, **d**), no CPZ iTBS (**c**, **e**), CPZ sham (**f**, **h**) and CPZ iTBS (**g**, **i**) mice. **j** The density of cFos^+^ neun^+^ neurons in M1 and V2 of no CPZ sham (n = 3), no CPZ iTBS (n = 4), CPZ sham (n = 4) and CPZ iTBS (n = 4) mice. Three-way ANOVA: no CPZ v CPZ F (1, 23) = 81.17, p < 0.0001; M1 v V2 F (1, 23) = 33.79, p < 0.0001; sham v iTBS F (1, 23) = 7.03, p = 0.14; (no CPZ v CPZ) x (M1 v V2) F (1, 23) = 7.17, p = 0.01; (no CPZ v CPZ) x (sham v iTBS) F (1, 23) = 2.04, p = 0.17; (M1 v V2) x (sham v iTBS) F (1, 23) = 2.50, p = 0.13; (no CPZ v CPZ) x (M1 v V2) x (sham v iTBS) F (1, 23) = 0.07, p = 0.79. **k**–**r** Confocal images of cFos (green) and PV (red) immunohistochemistry in M1 (**k**, **l**, **o**, **p**) and V2 (**m**, **n**, **q**, **r**) of no CPZ sham (**k**, **m**), no CPZ iTBS (**l**, **n**), CPZ sham (**o**, **q**) and CPZ iTBS (**p**, **r**) mice. **s** Density of cFos^+^ PV^+^ neurons in M1 and V2 of no CPZ sham (n = 3), no CPZ iTBS (n = 4), CPZ sham (n = 3) and CPZ iTBS (n = 3) mice. Three-way ANOVA: no CPZ v CPZ F (1, 18) = 187.7, p < 0.0001; M1 v V2 F(1,18) = 31.08, p < 0.0001; sham v iTBS F (1, 18) = 25.54, p < 0.0001; (no CPZ v CPZ) x (M1 v V2) F (1, 18) = 4.18, p = 0.056; (no CPZ v CPZ) x (sham v iTBS) F (1, 18) = 27.5, p < 0.0001; (M1 v V2) x (sham v iTBS) F (1, 18) = 1.34, p = 0.26; (no CPZ v CPZ) x (M1 v V2) x (sham v iTBS) F (1, 18) = 1.55, p = 0.23. Data are presented as mean ± SD. Bonferroni post-test: *p < 0.05, **p < 0.01, ****p < 0.0001. Scale bars represent 20 µm

In the healthy CNS, iTBS promotes new OL survival and maturation in M1 and V2 [29]. However, following CPZ demyelination, iTBS significantly increased the myelin load of new M1 but not V2 OLs. As the outcome of rTMS can be influenced by basal neuronal activity [48], we compared the expression of cFos within each region. CFos is an immediate early gene that is expressed in response to neuronal activity [49–54]. P60 C57BL/6 J mice were placed on the control or CPZ diet for 5 weeks, before receiving a single session of sham stimulation or iTBS. Mice were perfused 60–120 min later (Fig. 5a). Coronal brain cryosections containing M1 or V2 were processed to detect cFos and the neuronal marker neun (Fig. 5b-i). In healthy control mice, very few cFos^+^ neun^+^ cells were detected in M1 or V2 (Fig. 5b, c, j), and this was not altered by a single session of iTBS (Fig. 5d, e, j). Following demyelination, when the density of cFos^+^ neun^+^ cells was significantly elevated in M1 and V2, it again appeared unchanged by a single session of iTBS (Fig. 5f–j). As rTMS, delivered in an iTBS pattern, is most effective at modulating the activity of a subset of cortical neurons, particularly the activity of interneurons [55–58], we also examined the effect of CPZ demyelination and iTBS on the density of cFos^+^ PV^+^ interneurons in M1 and V2 (Fig. 5k–r). CPZ-demyelination significantly increased the density of cFos^+^ PV^+^ interneurons in both regions (Fig. 5s) but this effect was further exacerbated by iTBS (Fig. 5s). These data suggest that iTBS increased PV^+^ inhibitory neuron activity in the demyelinated cortex, and an increased proportion of the cFos^+^ neun^+^ cells identified in iTBS mice would be inhibitory rather than excitatory neurons. As the total density of active neurons is higher in V2 than M1, iTBS may take longer or be unable to overcome the imbalance in excitatory and inhibitory activity in V2, contributing to the different cellular response to iTBS in M1 and V2.

### Surviving OLs do not reliably express OLIG2 after 4 weeks on a CPZ diet

**Fig. 6:**
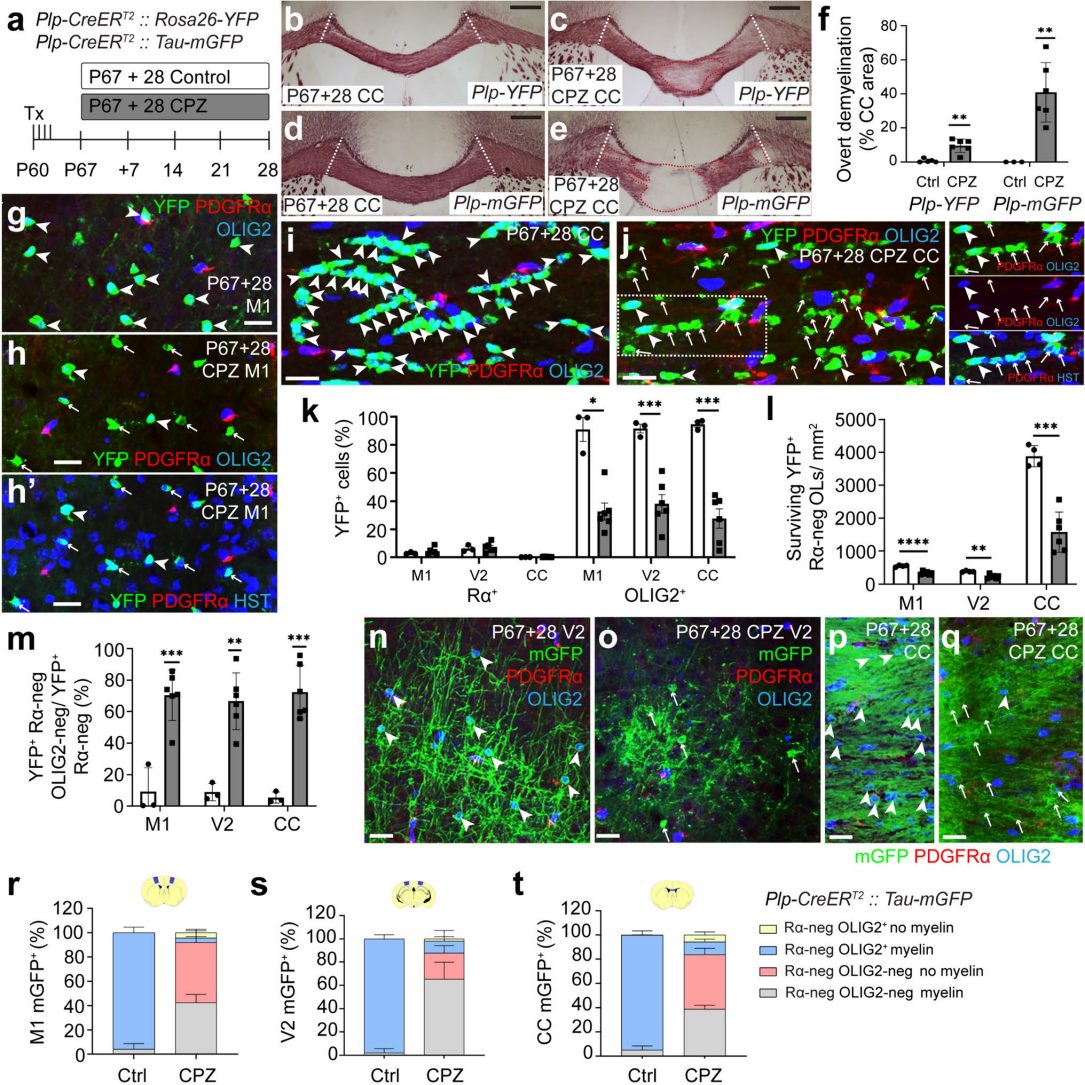
OLIG2 immunohistochemistry does not identify all cells of the OL lineage at 4 weeks of CPZ feeding. **a** Experimental schematic showing the timeline over which *Plp-CreER*^*T2*^*:: Rosa26-YFP* or *Plp-CreER*^*T2*^*:: Tau-mGFP* transgenic mice received 4 consecutive daily doses of Tx and up to 28 days of CPZ-feeding. **b**–**e** Images of black-gold labelling in the CC of P67 + 28 control and CPZ *Plp-CreER*^*T2*^*:: Rosa26-YFP* (**b**, **c**) or *Plp-CreER*^*T2*^*:: Tau-mGFP* mice (**d**, **e**). CC was analysed between the white dashed lines. Regions denoted by the red dashed lines show example regions of gross demyelination. **f** Area of the CC (%) where black-gold labelling was feint or absent in P67 + 28 control and CPZ *Plp-CreER*^*T2*^*:: Rosa26-YFP* or *Plp-CreER*^*T2*^*:: Tau-mGFP* mice. Data from each group was evaluated using a one sample t and Wilcoxon text to determine whether the level of demyelination was different from zero [n = 5 *Plp-CreER*^*T2*^*:: Rosa26-YFP* P67+28 controls, p = 0.19; n = 6 CPZ, p = 0.002; n = 3 *Plp-CreER*^*T2*^*:: Tau-mGFP* P67+28 controls (all zero); n = 6 CPZ, p = 0.002]. **g**–**j** Confocal images of YFP (green), PDGFRα (red) and OLIG2 (blue) immunohistochemistry in M1 (**g**, **h**) and CC (**i**, **j**) of P67+28 control (**g**, **i**) and CPZ (**h**, **j**) *Plp-CreER*^*T2*^*:: Rosa26-YFP* mice. Outset is the dashed box as separate colour channels. **h′** OLIG2 labelling for h. Arrowheads indicate YFP^+^ PDGFRα-neg OLIG2^+^ cells. Arrows indicate YFP^+^ PDGFRα-neg OLIG2-neg cells. **k** The proportion (%) of YFP^+^ cells that are PDGFRα^+^ OPCs or OLIG2^+^ cells in M1, V2 or the CC of *Plp-CreER*^*T2*^*:: Rosa26-YFP* P67+28 control (n = 3) or CPZ (n = 6) mice. Repeated measures two-way ANOVA with Geisser–Greenhouse correction for YFP^+^ PDGFRα^+^ [treatment F (1, 7) = 0.37, p = 0.56; region F (1.736, 12.15) = 23.67, p < 0.0001; interaction F (2, 14) = 0.24, p = 0.79] or YFP^+^ OLIG2^+^ [treatment F (1, 7) = 56.21, p = 0.0001; region F (1.28, 8.97) = 0.26, p = 0.68; interaction F (2, 14) = 0.77, p = 0.48]. **l** The density of YFP^+^ PDGFRα-neg surviving OLs in M1, V2 and CC of *Plp-CreER*^*T2*^*:: Rosa26-YFP* P67+28 control (n = 4) or CPZ (n = 6) mice. Repeated measures two-way ANOVA with Geisser–Greenhouse correction: treatment F (1, 8) = 48.42, p = 0.0001; region F (1.01, 8.1) = 225.2, p < 0.0001; interaction F (2, 16) = 45.84, p < 0.0001. **m** The proportion of YFP^+^ PDGFRα-neg cells that were OLIG2-neg in M1, V2 and the CC of *Plp-CreER*^*T2*^*:: Rosa26-YFP* P67+28 control (n = 3) and CPZ (n = 6) mice. Data from each group was evaluated using a one sample t and Wilcoxon text to determine whether any group was different from the expected value of zero, followed by a Benjamini–Hochberg correction for 6 comparisons: M1 *Plp-CreER*^*T2*^*:: Rosa26-YFP* P67+28 control, p = 0.41; CPZ, p = 0.0006; V2 *Plp-CreER*^*T2*^*:: Rosa26-YFP* P67+28 control, p = 0.11; CPZ, p = 0.002; CC *Plp-CreER*^*T2*^*:: Rosa26-YFP* P67+28 control, p = 0.12; CPZ, p = 0.0006. **n**–**q** Confocal images of mGFP (green), PDGFRα (red) and OLIG2 (blue) immunohistochemistry in V2 (**n**, **o**) or CC (**p**, **q**) of *Plp-CreER*^*T2*^*:: Tau-mGFP* P67+28 control (**n**, **p**) and CPZ (**o**, **q**) mice. Arrowheads indicate YFP^+^ PDGFRα-neg OLIG2^+^ OLs. Arrows indicate YFP^+^ PDGFRα-neg OLIG2-neg presumptive OLs. **r**–**t** Stacked column charts showing the proportion of mGFP^+^ PDGFRα-neg cells that are OLIG2^+^ or OLIG2-neg with or without myelin sheaths in M1 (**r**), V2 (**s**) and the CC (**t**) of *Plp-CreER*^*T2*^*:: Tau-mGFP* P67+28 control (n = 3) and CPZ (n = 3) mice. One sample t and Wilcoxon test with Benjamini–Hochberg correction for multiple comparisons determined whether the proportion of mGFP^+^ PDGFRα-neg OLIG2-neg cells was different from zero: M1 *Plp-CreER*^*T2*^*:: Tau-mGFP* P67+28 control, p = 0.27; CPZ, p = 0.003; V2 *Plp-CreER*^*T2*^*:: Tau-mGFP* P67+28 control, p = 0.423; CPZ, p = 0.03; CC *Plp-CreER*^*T2*^*:: Tau-mGFP* P67+28 control, p = 0.125; CPZ, p = 0.001. A repeated measures two-way ANOVA with Greenhouse–Geisser correction compared the proportion of mGFP^+^ PDGFRα-neg cells without myelin sheaths: treatment F (1, 4) = 7291, p < 0.0001; region F (1.42, 5.68) = 10.1, p = 0.02; interaction F (2, 8) = 10.04, p = 0.007. Data are presented as mean ± SD. **p < 0.01, ***p < 0.001. Scale bars represent 100 µm (b-e) or 20 µm (g-j, n-q)

Mature OLs that survive a demyelinating insult have the potential to contribute to myelin repair [59]. To fluorescently label mature OLs, follow their fate, and evaluate their response to iTBS, we administered Tx to P60 *Plp-CreER*^*T2*^*:: Rosa26-YFP* and *Plp-CreER*^*T2*^*:: Tau-mGFP* transgenic mice. Mice commenced CPZ feeding at P67 (Fig. 6a). Black-gold myelin labelling at P67+28CPZ (Fig. 6b-e) revealed that callosal demyelination was more pronounced in *Plp-CreER*^*T2*^*:: Tau-mGFP* mice compared with *Plp-CreER*^*T2*^*:: Rosa26-YFP* mice (Fig. 6f). However, CPZ-feeding successfully induced demyelination in both mouse cohorts. To evaluate YFP^+^ OL survival, we performed immunohistochemistry to detect YFP, PDGFRα and OLIG2 or ASPA (Fig. 6g–j). A very small subset of YFP^+^ cells were PDGFRα^+^ OLIG2^+^ OPCs in M1, V2 and the CC of P67+28 control and CPZ mice (Fig. 6k) and in controls > 91% of YFP^+^ cells in the M1, V2 and CC were OLIG2^+^ (Fig. 6g, i, k). CPZ feeding not only reduced the density of YFP^+^ cells in M1, V2 and the CC (Fig. 6l), but was associated with a high proportion of surviving YFP^+^ cells no longer labelling with the OLIG2 antibody (Fig. 6h, j, m). These YFP^+^ PDGFRα-neg OLIG2-neg cells had a small soma with minimal cytoplasm around the nucleus, consistent with the morphology of OLs (Fig. 6h, j) and comprised ~ 70% of M1 YFP^+^ cells, ~ 67% of V2 YFP^+^ cells and ~ 73% of CC YFP^+^ cells (Fig. 6m). As the vast majority of YFP^+^ cells in M1 (~ 91%) and V2 (~ 96%), and ~ 42% in the CC, still co-labelled with ASPA (Fig. S6), we considered these YFP^+^ cells to be surviving OLs.

We similarly performed immunohistochemistry on the more severely demyelinated P67+28 *Plp-CreER*^*T2*^*:: Tau-mGFP* control and CPZ mice (Fig. 6n–t). In the M1, V2 and CC of P67+28 control *Plp-CreER*^*T2*^*:: Tau-mGFP* mice, the vast majority of mGFP^+^ cells were PDGFRα-neg and OLIG2^+^ OLs that had the distinct morphology of myelinating OLs (Fig. 6n, p, r–t). CPZ feeding decreased the density of mGFP^+^ cells in M1 by ~ 79% and in the V2 and CC by ~ 66% (Fig. S7). However, following CPZ-feeding ~ 92% of M1 mGFP^+^ cells, ~ 88% of V2 mGFP^+^ cells, and ~ 84% of CC mGFP^+^ cells were PDGFRα-neg OLIG2-neg cells, and only a subset continued to support mGFP^+^ internodes (~ 45% of M1 mGFP^+^ cells; ~ 75% of V2 mGFP^+^ cells and ~ 50% of CC mGFP^+^ cells) (Fig. 6r–t). Despite this, essentially all mGFP^+^ cells co-labelled with ASPA (Fig. S7) and we considered these cells to be “surviving OLs”.

### iTBS delivery during CPZ feeding does not rescue pre-existing myelinating OLs

**Fig. 7:**
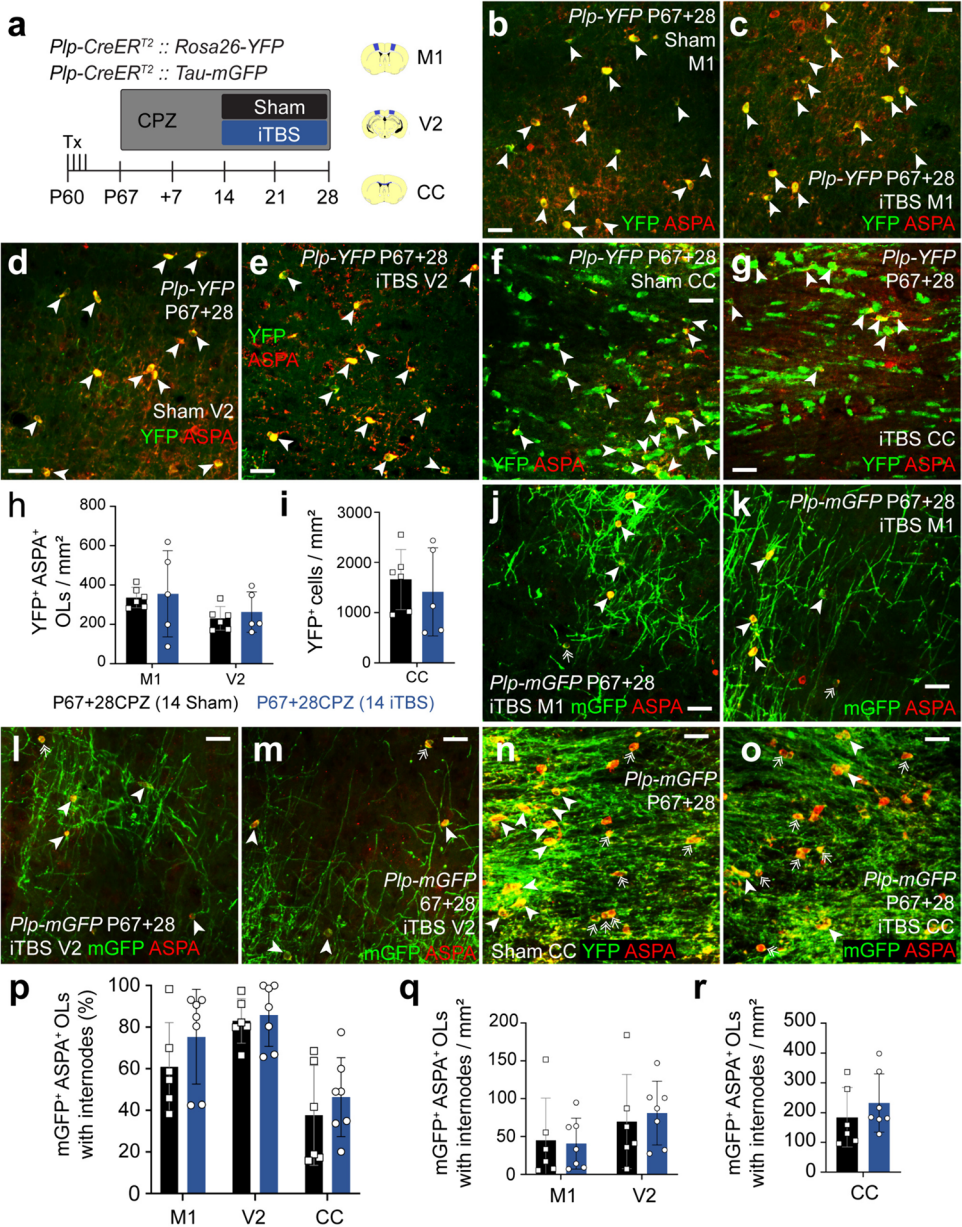
iTBS does not affect the density of surviving OLs that myelinate in the cortex or CC of CPZ-fed mice. **a** Schematic of timeline over which *Plp-CreER*^*T2*^*:: Rosa26-YFP* or *Plp-CreER*^*T2*^*:: Tau-mGFP* transgenic mice received 4 consecutive daily doses of Tx, 28 days of CPZ-feeding and 14 days of LI-rTMS (sham-stimulation or iTBS). **b**–**g** Confocal images of YFP (green) and ASPA (red) immunohistochemistry in M1 (**b**, **c**), V2 (**d**, **e**) or the CC (**f**, **g**) of *Plp-CreER*^*T2*^*:: Rosa26-YFP* P67+28 sham or iTBS mice. Arrowheads indicate surviving YFP^+^ ASPA^+^ OLs. **h** The density of YFP^+^ ASPA^+^ OLs in M1 and V2 of *Plp-CreER*^*T2*^*:: Rosa26-YFP* P67+28 sham (black, n = 6) or iTBS (blue, n = 5) mice. Repeated measures two-way ANOVA: treatment F (1, 9) = 0.15, p = 0.71; region F (1, 9) = 10.15, p = 0.01; interaction F (1, 9) = 0.045, p = 0.84. **i.** The density of YFP^+^ cells in the CC of *Plp-CreER*^*T2*^*:: Rosa26-YFP* P67 + 28 sham (black, n = 6) or iTBS (blue, n = 5) mice. Unpaired t-test, p = 0.59. **j**–**o** Confocal images of mGFP (green) and ASPA (red) immunohistochemistry in M1 (**j**, **k**), V2 (**l**, **m**) and the CC (**n**, **o**) of *Plp-CreER*^*T2*^*:: Tau-mGFP* P67+28 sham and iTBS mice. Arrowheads indicate mGFP^+^ ASPA^+^ OLs. **p** The proportion (%) of mGFP^+^ ASPA^+^ OLs with internodes in M1, V2 and CC of *Plp-CreER*^*T2*^*:: Tau-mGFP* P67+28 sham (black, n = 6) and iTBS (blue, n = 7) mice. Repeated measures two-way ANOVA with Geisser–Greenhouse correction: treatment F (1, 11) = 0.742, p = 0.41; region F (1.75, 19.28) = 49.87, p < 0.0001; interaction F (2, 22) = 0.89, p = 0.43. **q**–**r** The density of mGFP^+^ ASPA^+^ OLs with internodes in M1 and V2 (**q**) or the CC (**r**) of *Plp-CreER*^*T2*^*:: Tau-mGFP* P67+28 sham (black, n = 6) and iTBS (blue, n = 7) mice. Repeated measures two-way ANOVA with the Geisser–Greenhouse correction: treatment F (1, 11) = 0.29, p = 0.59; region F (1.2, 13.19) = 53.7, p < 0.0001; interaction F (2, 22) = 1.26, p = 0.3. Data are presented as mean ± SD. Scale bars represent 20 µm

To determine whether iTBS can alter the survival of mature OLs exposed to CPZ, Tx was administered to P60 *Plp-CreER*^*T2*^*:: Rosa26-YFP* and *Plp-CreER*^*T2*^*:: Tau-mGFP* transgenic mice. CPZ feeding commenced at P67, and from P67+14 mice received daily sham or iTBS (Fig. 7a). When we performed immunohistochemistry on coronal brain cryosections from P67+28CPZ *Plp-CreER*^*T2*^*:: Rosa26-YFP* sham or iTBS mice to detect YFP and ASPA (Fig. 7), we found that the density of surviving YFP^+^ ASPA^+^ OLs was equivalent in sham and iTBS mice [M1: sham ~ 336 cells/mm^2^ v iTBS ~ 355 cells/ mm^2^; V2 sham ~ 230 cells/mm^2^ v iTBS ~ 263 cells/mm^2^; CC sham ~ 1660 cells/mm^2^ v iTBS ~ 1415 cells/mm^2^; Fig. 7b–i). As CPZ can induce a dying-back process, whereby some of the myelin sheaths degenerate while the cell bodies remain [59, 60], we also determined whether surviving mGFP^+^ ASPA^+^ OLs supported internodes in M1, V2 or the CC (Fig. 7j–o). In sham mice, most surviving mGFP^+^ ASPA^+^ cortical OLs retained some internodes (sham stimulated: M1 ~ 13 OLs without internodes/mm^2^ vs. ~ 45 OLs with internodes/mm^2^; V2 ~ 10 OLs without internodes/mm^2^ vs ~ 70 OLs with internodes/mm^2^; Fig. S8). By contrast, most CC mGFP^+^ ASPA^+^ OLs lacked internodes (sham stimulated: ~ 364 OLs without internodes/mm^2^ vs. ~ 184 OLs with internodes/mm^2^; Fig. S8). Importantly, we found that the proportion and density of mGFP^+^ ASPA^+^ OLs with internodes was equivalent in sham and iTBS mice (Fig. 7j–r). These data indicate that 4 weeks of CPZ resulted in a higher proportion of mature OLs losing their myelin internodes in the CC relative to the cortex, but also indicated that delivering iTBS did not alter the number of “bare” OLs.

### iTBS increases the contribution that surviving OLs make to remyelination in the CC

**Fig. 8:**
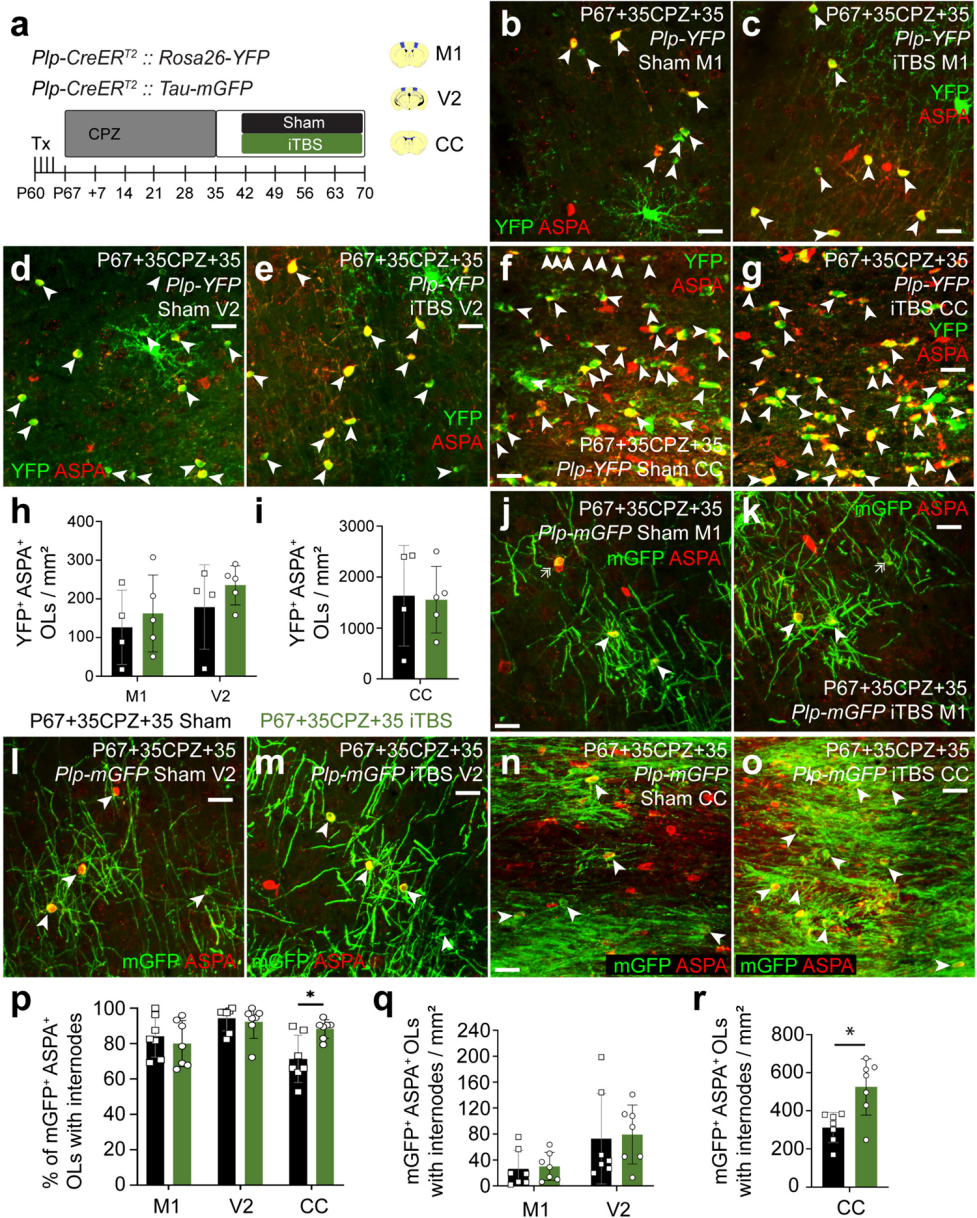
iTBS during remyelination increases the number of surviving myelinating OLs in the corpus callosum. **a** Experimental schematic showing the timeline over which *Plp-CreER*^*T2*^*:: Rosa26-YFP* or *Plp-CreER*^*T2*^*:: Tau-mGFP* transgenic mice received 4 consecutive daily doses of Tx, 35 days of CPZ-feeding and 28 days of LI-rTMS (sham-stimulation or iTBS). **b**–**g** Confocal images of YFP (green) and ASPA (red) immunohistochemistry in M1 (**b**, **c**), V2 (**d**, **e**) or the CC (**f**,** g**) of *Plp-CreER*^*T2*^*:: Rosa26-YFP* P67+35CPZ+35 sham (**b**, **d**, **f**) or iTBS (**c**, **e**, **g**) mice. Arrowheads indicate YFP^+^ ASPA^+^ OLs. **h**–**i** The density of YFP^+^ ASPA^+^ OLs in M1 and V2 (**h**) or the CC (**i**) of *Plp-CreER*^*T2*^*:: Rosa26-YFP* P67+35CPZ+35 sham (black, n = 4) or iTBS (green, n = 5) mice. Repeated measures two-way ANOVA with Geisser–Greenhouse correction: treatment F (1, 7) = 0.0003, p = 0.99; region F (1.011, 7.074) = 32.88, p = 0.0007; interaction F (2, 14) = 0.066, p = 0.9. **j**–**o** Confocal images of mGFP (green) and ASPA (red) immunohistochemistry in M1 (**j**, **k**), V2 (**l**, **m**) or the CC (**n**, **o**) of *Plp-CreER*^*T2*^*:: Tau-mGFP* P67+35CPZ+35 sham or iTBS mice. Arrowheads indicate mGFP^+^ ASPA^+^ OLs. **p** The proportion (%) of mGFP^+^ ASPA^+^ OLs with internodes in M1, V2 or the CC of *Plp-CreER*^*T2*^*:: Tau-mGFP* P67+35CPZ+35 sham (black, n = 7) or iTBS (green, n = 7) mice. Repeated measures two-way ANOVA with Geisser–Greenhouse correction: treatment F (1, 12) = 0.88, p = 0.37; region F (1.58, 18.92) = 8.65, p = 0.004; interaction F (2, 24) = 5.613, p = 0.01. **q**–**r** Quantification of the density of mGFP^+^ ASPA^+^ OLs with internodes in M1 and V2 (**q**) or the CC (**r**) of *Plp-CreER*^*T2*^*:: Tau-mGFP* P67+35CPZ+35 sham (black, n = 7) or iTBS (green, n = 7) mice. Repeated measures two-way ANOVA with Geisser–Greenhouse correction: treatment F (1, 12) = 6.55, p = 0.025; region F (1.15, 13.77) = 135.5, p < 0.0001; interaction F (2, 24) = 10.76, p = 0.0005. Data are presented as mean ± SD. *p < 0.05. Scale bars represent 20 µm

As iTBS can increase the myelin load of new M1 OLs during and after CPZ feeding, it may also support internode elaboration by OLs that survive a demyelinating event [12, 59]. To determine whether iTBS can increase the contribution that surviving OLs make to myelin repair, a second cohort of *Plp-CreER*^*T2*^*:: Rosa26-YFP* and *Plp-CreER*^*T2*^*:: Tau-mGFP* mice commenced CPZ feeding at P67, but were returned to normal chow from P67+35. One week later, they received the first of 28 consecutive daily sham or iTBS sessions (Fig. 8a). Coronal brain cryosections from P67+35CPZ+35 *Plp-CreER*^*T2*^*:: Rosa26-YFP* (Fig. 8b–i) and *Plp-CreER*^*T2*^*:: Tau-mGFP* mice (Fig. 8j–r) were processed to detect YFP or mGFP and ASPA. Essentially all YFP^+^ cells expressed ASPA, identifying them as surviving OLs. We noted that the density of YFP^+^ ASPA^+^ OLs in M1 fell significantly between P67+28CPZ and P67+35CPZ+35 (compare M1 sham data in Fig. 7h and Fig. 8h or see Fig. S8, p = 0.04) but was unchanged in V2 or the CC (compare Fig. 7h, i with Fig. 8h, i or see Fig. S8, p > 0.99). These data suggest that mature OLs die in M1 over a longer time-course than those in V2 or the CC. As the density of YFP^+^ ASPA^+^ OLs in M1, V2 or the CC was equivalent in sham and iTBS mice, iTBS did not modify the survival of YFP^+^ OLs after CPZ withdrawal (Fig. 8h, i).

By analysing the morphology of mGFP^+^ ASPA^+^ OLs in sham-stimulated *Plp-CreER*^*T2*^*:: Tau-mGFP* mice, we found that the proportion of OLs with internodes did not change significantly in M1 or V2 between P67+28CPZ and P67+35CPZ+35 (Fig. S8). By contrast, more surviving mGFP^+^ ASPA^+^ callosal OLs supported internodes in the CC of P67+35CPZ+35 mice relative to P67+28CPZ mice (compare sham data in Fig. 7p, r with Fig. 8p, r or see Fig. S8). This equated to ~ 184 mGFP^+^ ASPA^+^ OLs with internodes/mm^2^ at P67+28CPZ vs. ~ 313 mGFP^+^ OLs with internodes/ mm^2^ at P67+35CPZ+35. These data confirm that a subset of surviving callosal OLs spontaneously elaborate new myelin sheaths upon CPZ withdrawal. We found that delivering iTBS did not alter the proportion or density of mGFP^+^ OLs that elaborated internodes in M1 or V2 (Fig. 8p, q). However, iTBS significantly increased the proportion of mGFP^+^ OLs that had internodes in the CC (Fig. 8p). The density of myelinating mGFP^+^ OLs was increased by ~ 41%, relative to sham-stimulated mice (Fig. 8r). These data indicate that iTBS, delivered during remyelination enables more surviving callosal OLs to contribute to remyelination.

### iTBS increases the proportion of axons that are myelinated in M1 and the CC

**Fig. 9:**
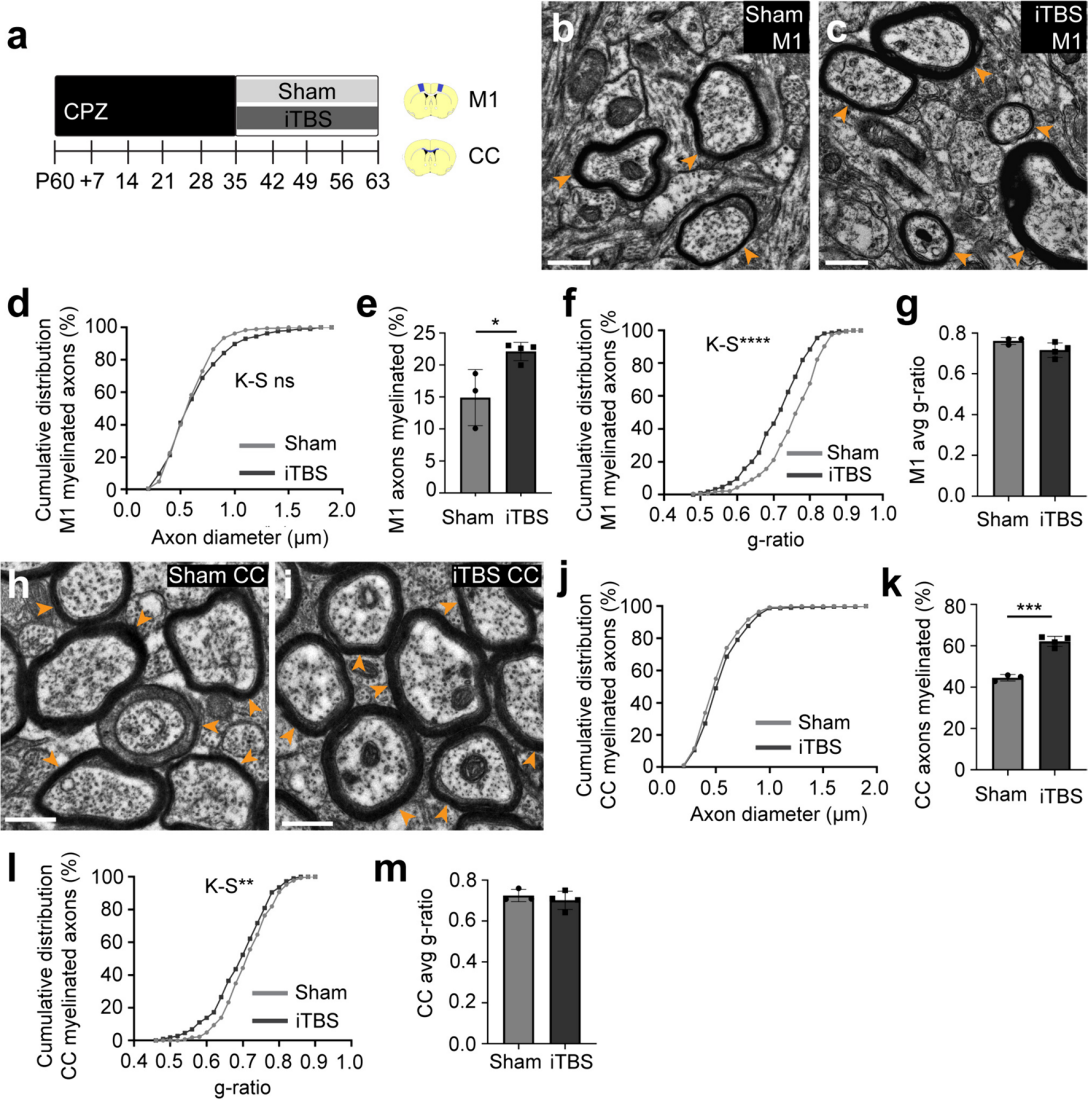
iTBS increases the proportion of axons that are myelinated in M1 and the CC. **a** P60 mice received 35 days of CPZ-feeding and commenced 28 days of LI-rTMS (sham-stimulation or iTBS) with CPZ withdrawal. **b**–**c** Transmission electron micrograph from M1 of a P60+35CPZ+28 sham (**b**) or iTBS (**c**) mouse. Orange arrowheads indicate examples of myelinated axons. **d** Cumulative distribution plot quantifying myelination relative to axon diameter in M1 of P60+35CPZ+28 sham (grey) or iTBS (black) mice [n = 185 sham and n = 215 iTBS axons, Kolmogorov–Smirnov (K–S) test D = 0.11, p = 0.21]. **e** The proportion (%) of M1 axons that are myelinated in P60+35CPZ+28 sham (n = 3) or iTBS (n = 4) mice [unpaired t-test, M1 p = 0.025]. **f** Cumulative distribution plot showing the g-ratios for M1 myelinated axons in P60+35CPZ+28 sham (grey) or iTBS (black) mice [n = 185 sham and n = 215 iTBS axons, Kolmogorov–Smirnov (K–S) test D = 0.27, p < 0.0001]. **g** Average M1 g-ratio per mouse for P60+35CPZ+28 sham (n = 3) and iTBS (n = 4) mice [unpaired t-test, p = 0.1]. **h**–**i** Transmission electron micrograph of the CC in a P60+35CPZ+28 sham (**h**) or iTBS (i) mouse. Orange arrowheads indicate example myelinated axons. **j.** Cumulative distribution plot showing the diameter of myelinated CC axons in P60+35CPZ+28 sham (grey) or iTBS (black) mice [n = 266 sham and n = 325 iTBS axons, Kolmogorov–Smirnov (K–S) test D = 0.09, p = 0.2]. **k** The proportion (%) of axons that are myelinated in the CC of P60+35CPZ+28 sham (n = 3) or iTBS (n = 4) mice [unpaired t-test, p = 0.0001]. **l** Cumulative distribution plot showing the g-ratio of myelinated CC axons in P60+35CPZ+28 sham (grey) or iTBS (black) mice [n = 266 sham and n = 325 iTBS axons, Kolmogorov–Smirnov (K–S) test D = 0.14, p = 0.006]. **m** Average CC g-ratio per mouse for P60+35CPZ+28 sham (n = 3) and iTBS (n = 4) mice [unpaired t-test, p = 0.44]. Data are presented as mean ± SD. *p < 0.05, **p < 0.01, ***p < 0.001, ****p < 0.0001. Scale bars represent 0.5 µm

As iTBS influenced M1 OL myelin load and had the combined effect of increasing new CC OL internode length and increasing the proportion of surviving CC OLs with internodes, we aimed to determine whether iTBS altered remyelination in M1 or the CC. P60 C57BL/6 J mice received 5 weeks of CPZ before it was withdrawn, and the mice commenced 28 consecutive days of sham stimulation or iTBS (P60+35CPZ+28; Fig. 9a). Mice were perfused 24 h after the final stimulation and ultrathin horizontal M1 or sagittal CC sections generated for transmission electron microscopy. In M1, transected axons were identified by the pattern of transected microtubules [61] and classified as unmyelinated or myelinated (Fig. 9b, c). iTBS did not alter the diameter of myelinated M1 axons (Fig. 9d) but increased the proportion of M1 axons that were myelinated (Fig. 9e). iTBS also produced a left-ward shift in the g-ratio cumulative distribution plot, suggesting that myelin is thicker in the M1 cortex following iTBS (Fig. 9f); however, this did not result in an increase in average M1 g-ratio per mouse (Fig. 9g). In the CC, myelinated axon diameter was similarly unaffected by iTBS (Fig. 9h-j), however, the proportion of CC axons that were myelinated increased (Fig. 9k). iTBS was also associated with a small left-ward shift in the cumulative distribution plot for CC myelinated axon g-ratio (Fig. 9l); however, this was insufficient to alter the average g-ratio for myelinated CC axons per mouse (Fig. 9m). These data indicate that iTBS promotes M1 and CC remyelination.

## Discussion

LI-rTMS, applied in an iTBS pattern, modifies internode extension by new and surviving OLs to enhance remyelination. When iTBS was delivered during CPZ feeding, it did not increase new OL number (Fig. 1) or enhance the survival of myelinating OLs (Fig. 6) but increased the number of internodes elaborated by new M1 OLs (Fig. 2). This effectively increased the myelin load of each new M1 OL by ~ 471 µm. When iTBS was delivered after CPZ withdrawal (during remyelination), it did not increase the addition of new OLs to M1, V2 or the CC (Fig. 3). Instead, iTBS significantly increased the length of the internodes produced by new M1 and callosal OLs (Fig. 4) and increased the density of surviving callosal OLs that supported internodes (Fig. 8). These cellular changes increased the proportion of axons that were remyelinated in M1 and the CC (Fig. 9). These cellular effects may be the result of iTBS increasing PV^+^ interneuron activity in the demyelinated mouse cortex (Fig. 5).

### iTBS does not promote new OL survival during or after CPZ feeding

When LI-rTMS was delivered during CPZ feeding (Fig. 1) or after CPZ withdrawal (Fig. 3), an equivalent number of new M1 and V2 OLs were detected in sham-stimulated and iTBS mice. ~ 85% of the new M1 OLs produced during CPZ feeding die [59] and iTBS delivery did not save new OLs from toxin-mediated cell death. However, even after CPZ withdrawal, iTBS did not alter new OL number in any of the brain regions examined (Fig. 3). As we commenced iTBS 21 or 49 days after Tx administration, our quantification of new YFP^+^ OLs inevitably included new OLs that differentiated prior to the LI-rTMS period. While this time-course diluted our capacity to detect even the established effect of iTBS on new OL number in the healthy CNS [29], it did not prevent it (Fig. 1). Therefore, we conclude that iTBS does not exert a gross effect on new OL number if delivered during CPZ demyelination or remyelination. As CPZ withdrawal is associated with rapid remyelination, it is possible that environment cues significantly enhance new OL survival [8, 62] and render the effect of iTBS on new OL survival redundant. However, it is also possible that the change in neuronal activity induced by demyelination (Fig. 5) alters the effect that LI-rTMS has on new OL survival.

### iTBS may reduce demyelination-induced cortical hyperactivity by activating PV^+^ interneurons

Our results suggest that iTBS can alter the balance of excitation and inhibition in the demyelinated cortex. It is well established that cortical activity is increased in dys and demyelinated mice: Shiverer mice experience epileptic seizures from 8 weeks of age [63, 64] and 4 weeks of CPZ feeding results in interictal epileptiform discharges in the mouse neocortex [65], indicative of the hypersynchronized burst firing of projection and interneurons [66, 67]. In our study, this change in activity was detected as an increase in cFos^+^ neun^+^ cell density in M1 and V2 at 5 weeks of CPZ feeding (Fig. 5). A single session of iTBS did not increase the overall density of active neurons, but significantly increased the density of active fast spiking PV^+^ interneurons.

PV^+^ interneurons are primarily basket cells that are reciprocally connected to excitatory projection neurons and other interneurons, allowing them to provide fast and temporally precise inhibitory regulation [68–71]. The optogenetic modulation of PV^+^ interneurons can suppress or enhance cortical epileptiform activity [72–75], but in the cortex of CPZ demyelinated mice, the optogenetic activation of PV^+^ interneurons reduces the frequency of interictal epileptiform events [65]. As a single session of iTBS significantly increased the density of cFos^+^ PV^+^ interneurons in the M1 and V2 cortices, it is likely that iTBS acts to counteract the demyelination-induced cortical hyperactivity. Our results match those of others showing that PV^+^ interneurons are strongly modulated by rTMS [55, 58, 76–78] although the direction of modulation is highly dependent on TMS intensity, frequency and number of sessions, as well as brain state - for example the effect can be different in awake versus anaesthetised rodents [53]. As PV^+^ interneurons also regulate brain wide oscillations [65, 79–81], they likely mediate the effects of LI-rTMS on brain network synchronisation in rodents [57] and theta oscillations in humans [82, 83]. Therefore, we propose that iTBS alters the activity of PV^+^ interneurons and promotes remyelination, however, it is unclear whether the restoration of normal network activity supports remyelination or visa versa. It will be important in future studies to examine the activity of PV^+^ interneurons across the time-course of iTBS delivery, to determine when and how cortical activity is modified relative to myelin repair.

### iTBS increases the myelin load of new OLs in M1 but not V2

Grey matter demyelination can be extensive in the brains of people with MS, often exceeding the more studied white matter demyelination [84, 85]. A high cortical lesion load is associated with physical disability and cognitive dysfunction [86, 87], making cortical remyelination an important therapeutic objective for MS research. When iTBS was delivered during CPZ feeding and for the first 7 days following CPZ withdrawal, it increased the number of internodes elaborated by new M1 OLs (Fig. 2). When we instead started iTBS 7 days after CPZ withdrawal, it had no effect on the number of internodes elaborated by new M1 OLs (Fig. 4). Instead, it increased the length of internodes elaborated by new M1, V2 and CC OLs (Fig. 4). This represented a small increase in average M1 OL internode length (~ 8 µm), but an estimated ~ 645 µm more myelin being synthesised per M1 OL (Fig. 4) - and was sufficient to increase the number of M1 axons being myelinated (Fig. 9). While remyelination can restore action potential saltatory conduction to projection neurons [88], the final conduction velocity is impacted by multiple parameters including axon diameter, the distribution of internodes along the axon, internode length, myelin thickness, node of Ranvier length, and periaxonal space width [89]. When delivered during remyelination, iTBS produced a relatively small increase in M1 and CC internode length (Fig. 4). This increase in internode length would be predicted to increase action potential conduction velocity, as the lengthened internodes were still shorter than the “flat maximum” - the length beyond which longer internodes would cause conduction velocity to decline [90, 91]. Furthermore, increasing myelin thickness can also increase action potential conduction velocity [92] and we report that iTBS shifts the g-ratio of myelinated M1 and CC axons towards smaller g-ratios, indicative of thicker myelin (Fig. 9).

It is unclear why iTBS is more effective at promoting internode extension by new OLs in M1 compared to V2, when it affects new OL in both regions of the healthy brain [29]. This could result from regional differences in the severity of OL death and demyelination, cortical organisation, or neuronal activity. CPZ does not produce a consistent level of demyelination along the rostro-caudal axis of the CC, with the rostral region generally experiencing less demyelination than the caudal region [45, 46]. It is unclear whether this is also true in the cortex, however, we found that 4 weeks of CPZ delivery to *Plp-CreER*^*T2*^*:: Rosa26-YFP* mice killed ~ 39% of the YFP^+^ PDGFRα-neg OLs in M1 and ~ 40% in V2 (Fig. 6), indicating the initial loss of mature OLs induced by CPZ was equivalent in M1 and V2 cortices. However, we also found that OL loss continued in M1 between P67+28CPZ and P67+35CPZ+35 mouse cohorts, consistent with a previous study of CPZ-induced OL death in M1 [59], but OL density remained stable over this time-course in V2 (Fig. S8). Additionally, after 4 weeks of CPZ feeding, only ~ 46% of surviving M1 OLs retained internodes, while ~ 76% of V2 OLs elaborated internodes (Fig. 6). Therefore, it is possible that the outcome of iTBS is impacted by the different time-course of OL loss and demyelination in M1 and V2.

rTMS can modulate neuronal activity in the brain [52, 57], however, the outcome of rTMS protocols is heavily influenced by the baseline level of neuronal activity within the targeted region [93]. Structural differences between M1 and V2 cortical circuitry [94] may influence the neuronal response to demyelination. Indeed, demyelination was associated with a significant increase in the density of cFos^+^ neun^+^ and cFos^+^ PV^+^ neurons in each region (Fig. 5), however, the higher density in V2 suggested that neuronal activity was more dysregulated in V2. A previous study reported that 2 weeks of 10 Hz rTMS reduced the density of cFos^+^ PV^+^ neurons [57], but in the demyelinated M1 and V2 cortices, a single session of iTBS increased the density of cFos^+^ PV^+^ neurons (Fig. 5), indicating that iTBS acutely promoted inhibitory neuronal activity in both regions. If consecutive sessions of iTBS can normalise activity in M1, this could promote remyelination, as sustained hyperactivity is known to negatively impact learning-induced remyelination [59]. Median neuronal firing rate increases by ~ 70% in the motor cortex in the week following CPZ-withdrawal but returns to normal by 3 weeks of remyelination [59]. Motor learning interventions that were ineffective during the period of hyperactivity instead increased the proportion of myelin sheathes that faithfully replaced lost internodes when delivered after activity returned to normal [59]. Not only are such regional and temporal differences in baseline GABAergic or glutamatergic signalling likely to influence the outcome of iTBS, but the modulation of GABAergic or glutamatergic signalling could account for the longer internodes that we observed [43, 95].

### iTBS supports internode generation by surviving OLs

A higher proportion of surviving OLs elaborate internodes in the CC of P67+35CPZ+35 mice following iTBS (Fig. 8). This could be the result of iTBS preserving internodes or supporting internode regeneration. If iTBS promoted internode preservation, it could only maintain but not increase the density of surviving OLs that myelinate over time. We determined that the density of surviving myelinating callosal OLs did not plateau, but rather increased between P67+28CPZ and P67+35CPZ+35 (Fig. S8) - indicating that iTBS must support surviving OLs to generate new remyelinating internodes.

Researchers have taken a variety of experimental approaches to gather evidence in support of OLs surviving a demyelinating injury and regenerating internodes [9, 12, 59, 96, 97]. In a feline model, spinal cord demyelination increases the variability of the g-ratios for axons myelinated by individual ventral column OLs - suggesting the OLs support some original and remyelinating sheathes [12]. The birth-dating of OLs within MS shadow-plaques, which are traditionally considered partially remyelinated lesions, also suggests that surviving OLs contribute to human remyelination [9]. However, definitive evidence of surviving OLs being able to grow new internodes has come from live imaging studies that followed OLs in the zebrafish spinal cord or mouse cortex during demyelination and remyelination [59, 96, 98]. In the zebrafish spinal cord, surviving OLs generated a small number of new internodes, but these frequently and inappropriately myelinated neuronal cell bodies [96]. In the mouse cortex, 3 weeks after CPZ-withdrawal, surviving OLs were more likely to remyelinate the denuded axons than new OLs [59]. However, under more inflammatory conditions, only a very small proportion of surviving cortical OLs elaborated new internodes [98]. Perhaps unsurprisingly, clemastine, which acts on OPCs to enhance remyelination, did not increase remyelination by surviving OLs [98]. However, motor learning, delivered 10–17 days after CPZ withdrawal, dramatically increased the proportion of surviving OL that made new myelin sheathes [59]. More research is required to determine how surviving OLs mechanistically extend new processes and wrap new sheathes, and how this is supported by iTBS. However, one possibility is that iTBS can promote extracellular signal-regulated kinases 1 and 2 (ERK1/2) phosphorylation [99], which is known to increase the number of surviving OLs that ensheath demyelinated axons [97].

### LI-rTMS as a remyelinating therapy for people with MS

LI-rTMS may be useful as an adjunct to immune modulatory therapy for the treatment of MS. Drugs or interventions that increase remyelination remain an unmet need for people with MS, as they hold the potential to restore neuron function, promote neuroprotection by limiting neurodegeneration, and increase functional recovery [100, 101]. We report that LI-rTMS, delivered in an iTBS pattern, can increase remyelination following CPZ-induced demyelination, by affecting the behaviour of new and surviving OLs. However, the outcome is likely to depend on the timing of LI-rTMS delivery relative to lesion development, the baseline neuronal activity in the targeted region, and the level of inflammation within the CNS. It was recently shown that surviving OLs in the inflamed and demyelinated cortex elaborate a small number of internodes that are abnormally short [98]. The CPZ-model of demyelination is associated with increased astrogliosis and microgliosis [102] but is not a model of inflammatory demyelination. Therefore, it would be interesting to determine whether LI-rTMS can improve the ability of surviving OLs to contribute to repair under more overtly inflammatory conditions. In people with MS, immune modulatory drugs can reduce the frequency of clinical relapses and impede peripheral immune cell egress into the CNS, however, treatments are still required that will prevent or reverse neuroinflammation, in the form of astrogliosis and microgliosis, which is associated with more severe disease progression [103, 104]. Several lines of evidence indicate that rTMS can direct astrocytes and microglia to transition from a pro-inflammatory to an anti-inflammatory phenotype when delivered following a CNS injury [105–111]. LI-rTMS can exert a cell autonomous effect on astrocytes, as a single session delivering 600 pulses (1 or 10 Hz, 18mT) downregulated the expression of proinflammatory genes in cultured mouse primary astrocytes [112]. The capacity for LI-rTMS to jointly suppress neuroinflammation and promote remyelination, suggests that it could be beneficial for people with MS on multiple fronts, however our studies also suggest that care will need to be taken to time LI-rTMS delivery to maximise its ability to promote myelin repair, and that this could fluctuate across the disease course.

### Considerations when using immunohistochemistry to quantify OL loss in mouse models of demyelination

The basic helix-loop-helix transcription factor, OLIG2, is expressed by cells of OL lineage [113]. The anti-OLIG2 antibody used in this study is sensitive to tissue fixation conditions but can be used to reliably identify cells of the OL lineage in the healthy adult mouse brain. However, our data suggest that there is a brief window during CPZ-feeding where OLIG2 labelling is poor, and the antibody cannot be used to accurately quantify surviving OL number. By tracing mature OLs during CPZ feeding, we determined that after 4 weeks of CPZ feeding a significant proportion of OLs within the cortex or CC could not be identified by OLIG2 labelling alone (Fig. 6). This issue was restricted to surviving OLs, as OLIG2 was clearly visible in PDGFRα^+^ OPCs and newborn OLs. Some of the surviving OLIG2-neg OLs lacked internodes, suggesting that they had undergone dying-back oligodendrogliopathy [60, 114], while others retained some myelin internodes. OLIG2 expression was recovered by all surviving OLs following CPZ withdrawal. We find this observation noteworthy as the fluorescently labelled OLs would have been assumed dead if we had completely relied on OLIG2 labelling. Our data suggest that ASPA identifies a higher proportion of the surviving OLs than OLIG2 during CPZ-feeding and may indicate that OL loss has been overestimated in studies that relied heavily on immunohistochemical readouts. The transient loss of OLIG2 immunohistochemistry may be functionally significant, as OLIG2 regulates OL differentiation and myelination [113, 115] and the genetic ablation of OLIG2 increases p53 activation leading to neural progenitor and OPC death [116]. As the activation of p53 can mediate OL apoptosis in CPZ-fed mice [117], it is possible that reduced OLIG2 expression precedes the p53-dependent apoptotic death of OLs in the CPZ model.

### Supplementary Information

Below is the link to the electronic supplementary material.

Supplementary file1 (DOCX 8599 KB)

## Data Availability

The image files and data described in this manuscript can be obtained from the corresponding author by reasonable request.
