# Supplementary material for: Low intensity repetitive transcranial magnetic stimulation enhances remyelination by newborn and surviving oligodendrocytes in the cuprizone model of toxic demyelination
Source: Cell Mol Life Sci. 2024 Aug 12;81(1):346. doi: 10.1007/s00018-024-05391-0 (PMC11335270; doi:10.1007/s00018-024-05391-0)
Supplement: Supplementary file 1 — Supplementary file1 (DOCX 8599 KB) [file 18_2024_5391_MOESM1_ESM.docx]

**Nguyen et al. Low intensity repetitive transcranial magnetic stimulation enhances remyelination by newborn and surviving oligodendrocytes in the curpizone model of toxic demyelination.**

**Supplementary Figures**


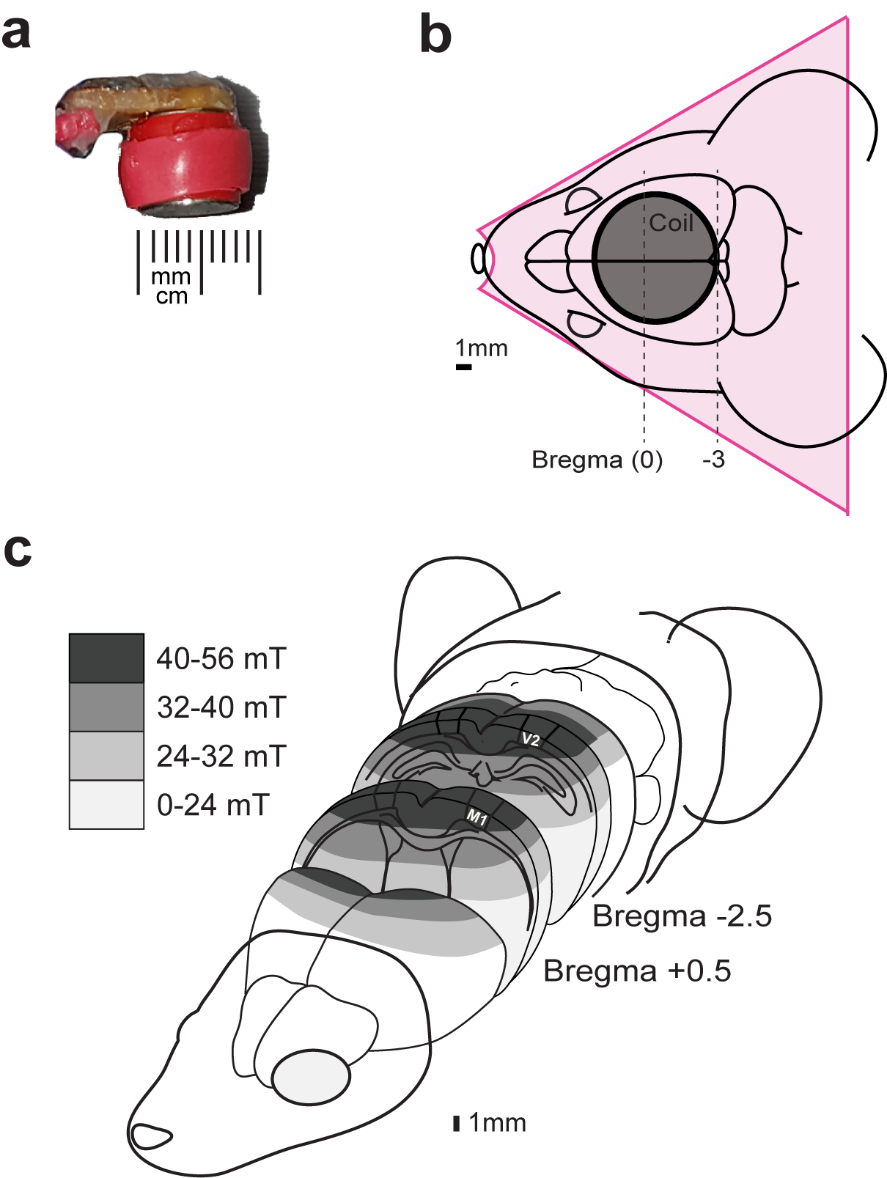


***Figure S1: LI-rTMS delivery to mice.***

**a.** A custom-built circular coil (8 mm outer diameter) was used to deliver LI-rTMS to mice [33]. **b**. Schematic showing the coil position relative to the mouse brain. The back of the LI-rTMS coil is aligned with the front of the ears at ~Bregma -3. The mouse is positioned within a plastic body-contour shape restraint cone (pink) during stimulation. **c.** Schematic illustration of estimated magnetic field intensity (mT) and the range of field intensities in brain regions of interest: M1 and CC at ~Bregma +0.5 and V2 at ~Bregma -2.5. Note that the magnetic field intensity decreases with increasing distance from the LI-rTMS coil.

**
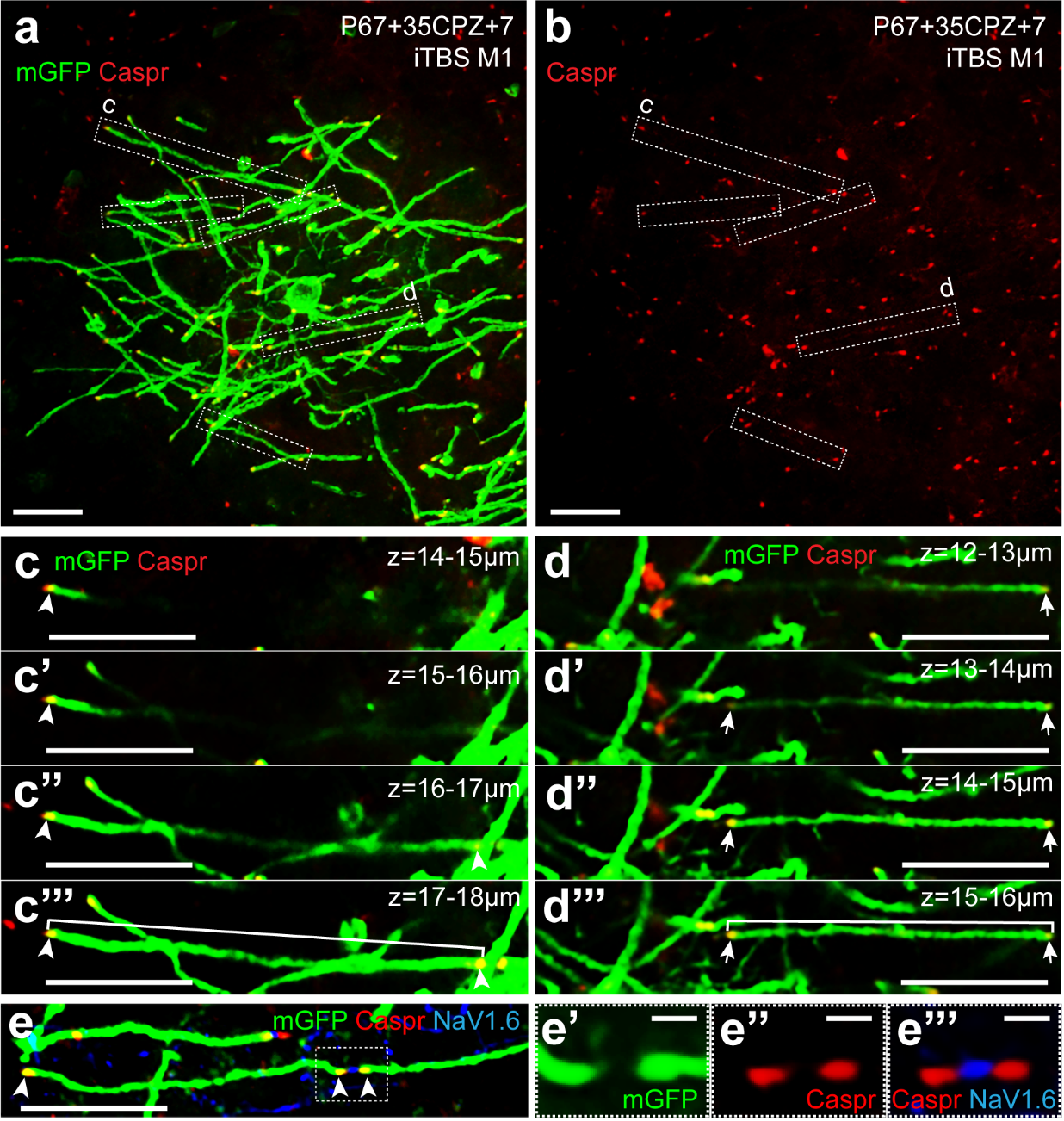
**

***Figure S2: Identifying intact internodes elaborated by new OLs in Pdgfrα-CreERT^T2^ :: Tau-mGFP mice***

**a-b.** Compressed confocal images showing mGFP^+^ internodes (green) and Caspr^+^ paranodes (red) in M1 of a P67+35CPZ+7 *Pdgfrα-CreERT^T2^ :: Tau-mGFP* mouse that received 4 doses of Tx from P60, commenced 35 days of CPZ feeding at P67 and commenced 28 days of iTBS from 14 days of CPZ (experimental schematic in Fig. 1a). Dashed boxes highlight example mGFP^+^ internodes flanked by Caspr^+^ paranodes. Note that mGFP and Caspr co-label the paranodes. **c-d.** Four consecutive z-planes through an intact mGFP^+^ internode allows it to be traced from one mGFP^+^ Caspr^+^ paranode to the other. **e.** Compressed confocal image showing two adjacent mGFP^+^ internodes (green), terminating at Caspr^+^ paranodes (red) and flanking a NaV1.6^+^ node of Ranvier (blue) Scale bars represent 20 µm (e) or 2 µm (e’-e’’’).

**
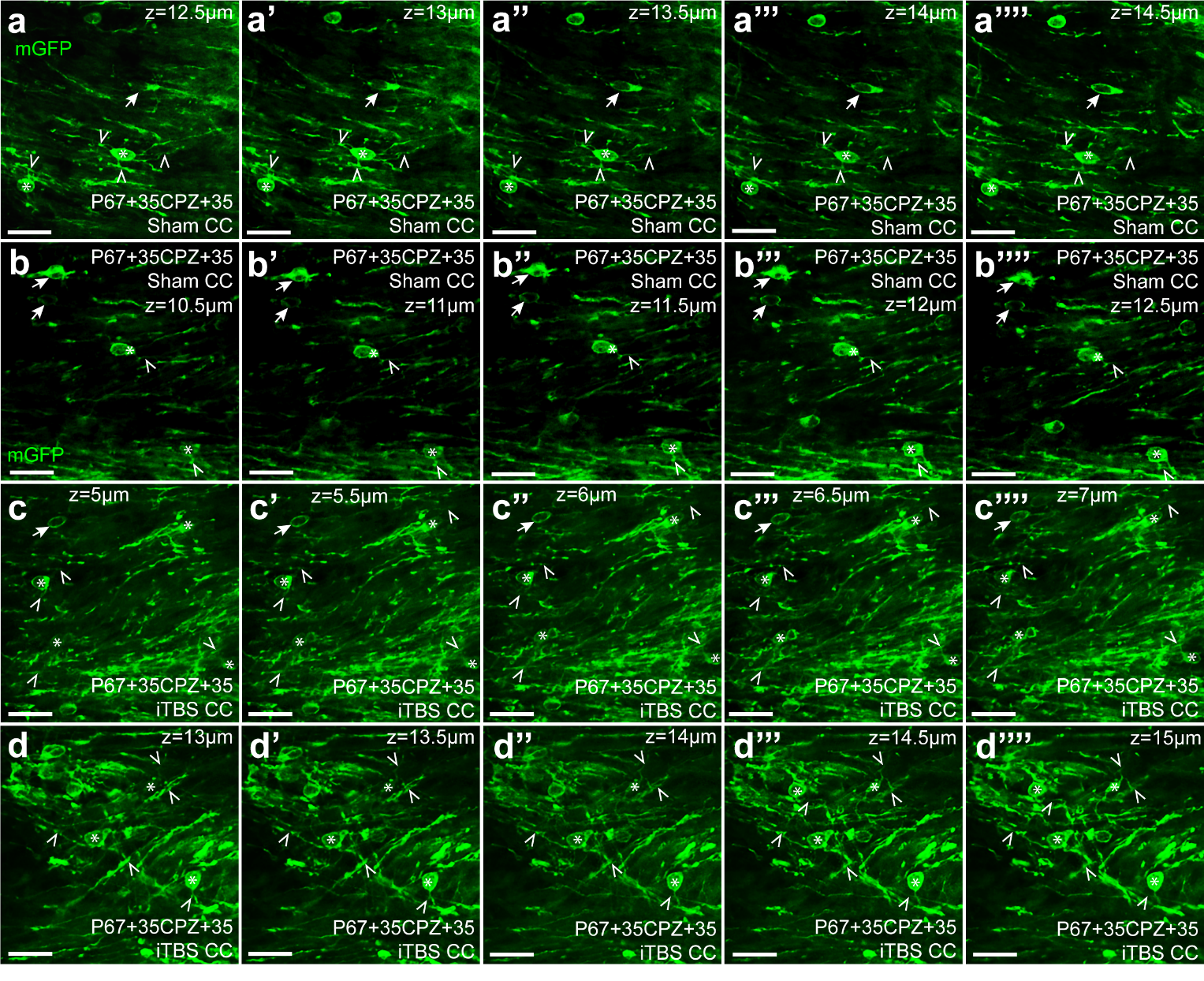
**

***Figure S3:* *A subset of* *mGFP OLs support internodes in the CC of P67+35CPZ+35 Plp-CreER^T2^ :: Tau-mGFP sham and iTBS mice.***

**a-d.** Each row shows five consecutive z-plane images (0.5 µm z-steps; 2 µm total) through surviving mGFP^+^ (green) OLs in the CC of P67+35CPZ+35 *Plp-CreER^T2^ :: Tau-mGFP* Sham (**a, b**) or iTBS (**c, d**) mice. The experimental time course is provided in Fig. 8a. By moving through z-planes that contained or were immediately adjacent to the OL soma, it was possible to determine whether the OL extended mGFP^+^ processes to one or more long, thin mGFP^+^ segments (presumptive internodes). mGFP^+^ OL soma that are not connected to mGFP^+^ internodes are denoted by a white arrow. mGFP^+^ OL soma that extend processes to mGFP^+^ internodes are marked with a white asterisk and their associated internodes indicated by a white arrowhead. After evaluating each mGFP^+^ soma, quantification was binary i.e. OLs were scored as having no internodes or having ≥ 1 internode. Internode number was not quantified. Scale bars represent 20 µm.


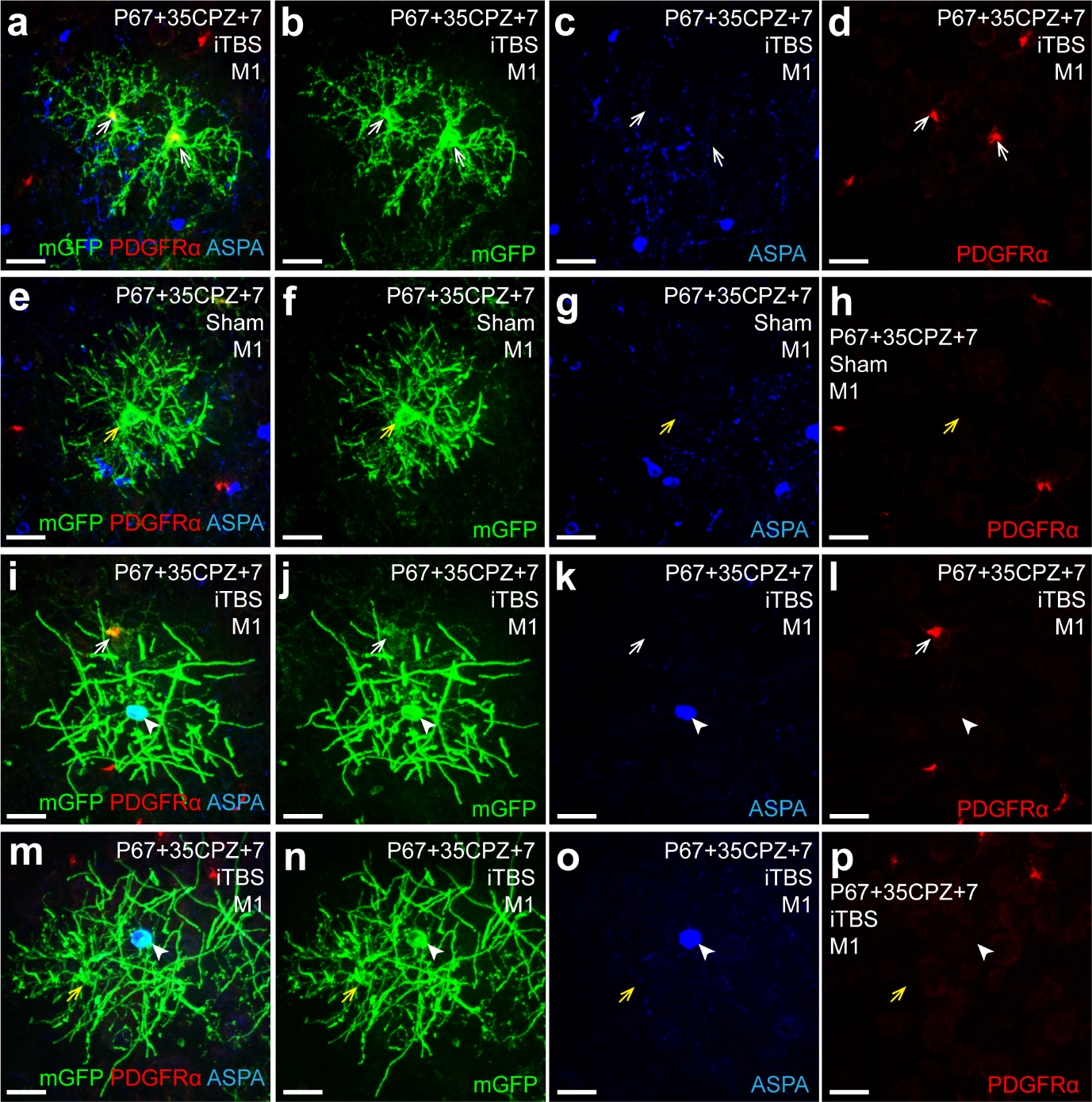


***Figure S4: mGFP^+^ OPCs generate ASPA^+^ mGFP myelinating OLs in Pdgfrα-CreERT^T2^ :: Tau-mGFP mice***

**a-d.** Maximum projection image of two PDGFRα^+^ ASPA-neg mGFP OPCs (white arrows) in M1 of a P67+35CPZ+7 *Pdgfrα-CreERT^T2^ :: Tau-mGFP* mouse that received 28 days of iTBS from 14 days of CPZ. **e-h.** Maximum projection image of a PDGFRα-neg ASPA-neg mGFP premyelinating OL (yellow arrow) in M1 of a P67+35CPZ+7 mouse that received sham stimulation. **i-l.** Maximum projection image of a PDGFRα-neg ASPA^+^ mGFP myelinating OL (arrowhead) and PDGFRα^+^ ASPA-neg OPC (arrow) in M1 of a P67+35CPZ+7 mouse that received iTBS. **m-p.** Maximum projection image of a mGFP premyelinating OL (yellow arrow) and mGFP myelinating OL (*Pdgfrα*-neg ASPA^+^, arrowhead) in M1 of a P67+35CPZ+7 mouse that received iTBS. Scale bars represent 20 µm.


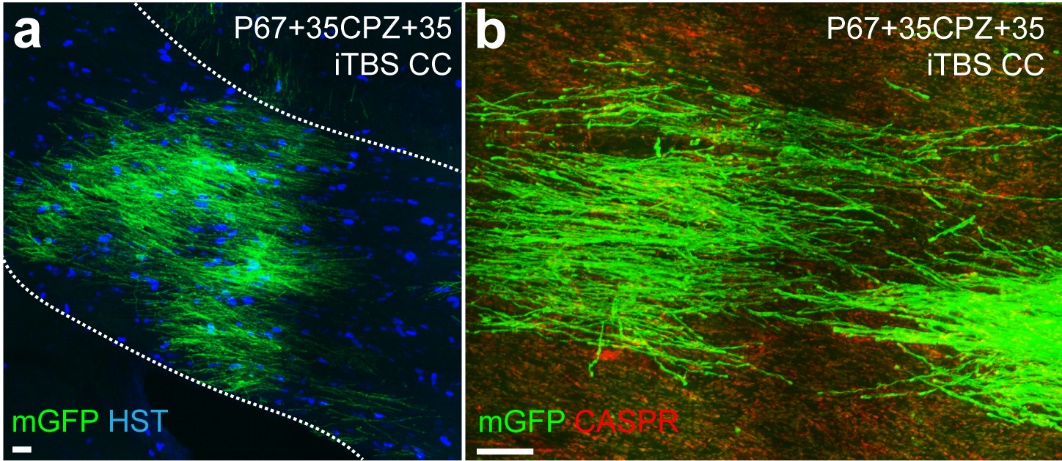


***Figure S5: P67+35CPZ+35 Pdgfrα-CreERT^T2^ :: Tau-mGFP mice have a high density of mGFP^+^ internodes in the CC***

**a, b.** Maximum projection images of mGFP (green) and HST (blue) (**a**) or mGFP (green) and CASPR (red) (**b**) in the CC of a P67+35CPZ+35 *Pdgfrα-CreERT^T2^ :: Tau-mGFP* mouse that commenced 28 consecutive daily sessions of iTBS 7 days after CPZ withdrawal. It was possible to discern individual mGFP^+^ internodes that were flanked by CASPR paranodes, but it was not possible to accurately determine which mGFP^+^ internodes belonged to a specific mGFP^+^ OL soma, due to the high density of new OLs and internodes. Scale bars represent 20 µm.


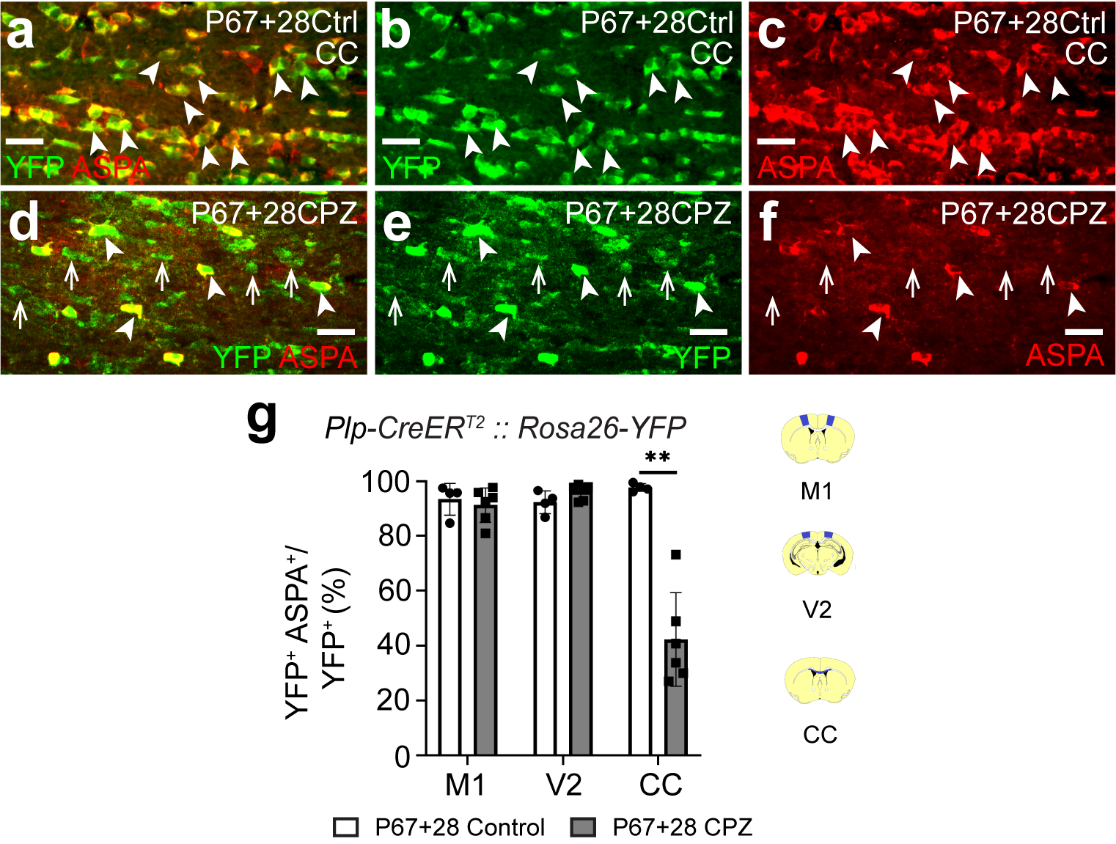


***Figure S6: A high proportion of YFP^+^ cells no longer co-express ASPA in the CC of P67+28 CPZ Plp-CreER^T2^ :: Rosa26-YFP mice.***

**a-f.** Confocal images of YFP (green) and ASPA (red) immunohistochemistry in the CC of P67+28 control (**a-c**) and CPZ (**d-f**) *Plp-CreER^T2^ :: Rosa26-YFP* mice. Arrowheads denote YFP^+^ ASPA^+^ OLs. Arrows denote YFP^+^ cells that do not express ASPA. **g.** The proportion (%) of YFP^+^ cells that co-express ASPA in M1, V2 and the CC of P67+28 *Plp-CreER^T2^ :: Rosa26-YFP* control (n=4) or CPZ mice (n=6). Repeated measures two-way ANOVA with Geisser-Greenhouse correction: treatment F (1, 8) = 29.3, p = 0.0006; region F (1.43, 11.43) = 22.65, p = 0.0002; interaction F (2, 16) = 33.33, p < 0.001. Schematics of coronal brain sections show the analysed regions in blue i.e. M1 (~ Bregma +0.5), V2 (~ Bregma -2.5) and CC underlying M1 (~ Bregma +0.5). Data are presented as mean ± SD. Bonferroni post-test: **p < 0.01. Scale bars represent 20 µm.


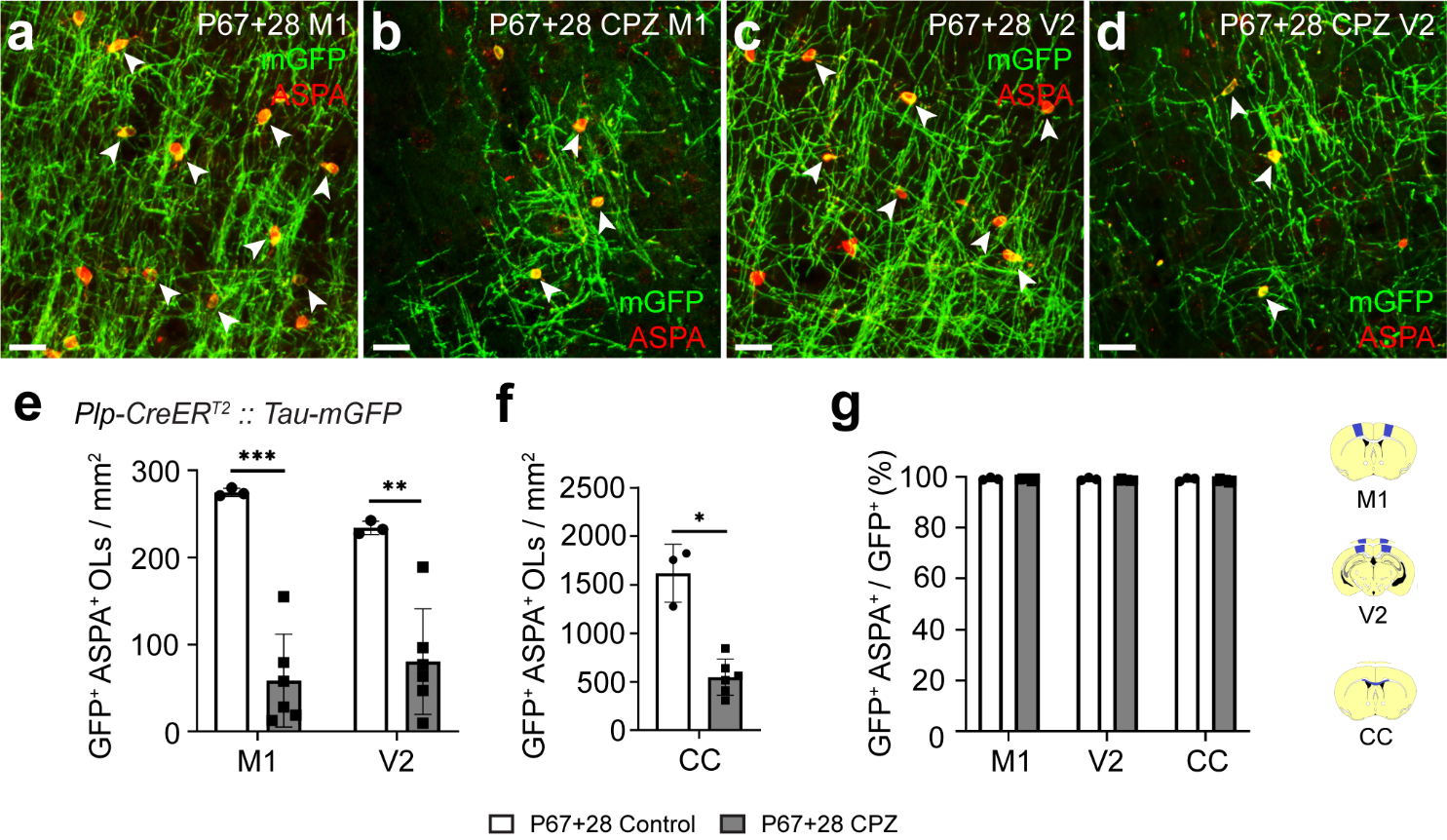


***Figure S7: CPZ-feeding reduced the density of mGFP^+^ OLs in M1, V2 and CC of Plp-CreER^T2^ :: Tau-mGFP mice.***

**a-d.** Confocal images of mGFP (green) and ASPA (red) immunohistochemistry in M1 (**a, b**) and V2 (**c, d**) of *Plp-CreER^T2^ :: Tau-mGFP* P67+28 control (**a, c**) and CPZ (**b, d**) mice. Arrowheads indicate mGFP^+^ ASPA^+^ OLs. **e-f.** The density of mGFP^+^ cells (OLs) in M1, V2 (**e**) and the CC (**f**) of P67+28 *Plp-CreER^T2^ :: Tau-mGFP* control (n=3) and CPZ mice (n=6). Repeated measures two-way ANOVA with Geisser-Greenhouse correction: treatment F (1, 7) = 79.68, p < 0.0001; region F (1.215, 8.5) = 120.7, p < 0.0001; interaction F (2, 14) = 27.87, p < 0.0001. **g.** The proportion of mGFP^+^ cells that co-label for ASPA in M1, V2 and CC of P67+28 *Plp-CreER^T2^ :: Tau-mGFP* (n=3) and P67+28CPZ *Plp-CreER^T2^ :: Tau-mGFP* (n=6) mice. Unlike P67+28CPZ *Plp-CreER^T2^ :: Rosa26-YFP* mice (Fig. S6), we do not see a significant population of mGFP^+^ that failed to label for ASPA in P67+28CPZ *Plp-CreER^T2^ :: Tau-mGFP* mice. This is likely due to this cell population having died already, due to the more severe demyelination that CPZ feeding produced in the P67+28CPZ *Plp-CreER^T2^ :: Tau-mGFP* mice (Fig. 6). Repeated measures two-way ANOVA with Geisser-Greenhouse correction: treatment F (1, 7) = 2.119, p = 0.19; region F (1.25, 8.74) = 1.52, p = 0.26; interaction F (2, 14) = 0.25, p = 0.78. Schematics of coronal brain sections show the analysed regions in blue i.e. M1 (~ Bregma +0.5), V2 (~ Bregma -2.5) and CC underlying M1 (~ Bregma +0.5). Data are presented as mean ± SD. Bonferroni post-test: *p < 0.05, **p < 0.01, ***p < 0.001.


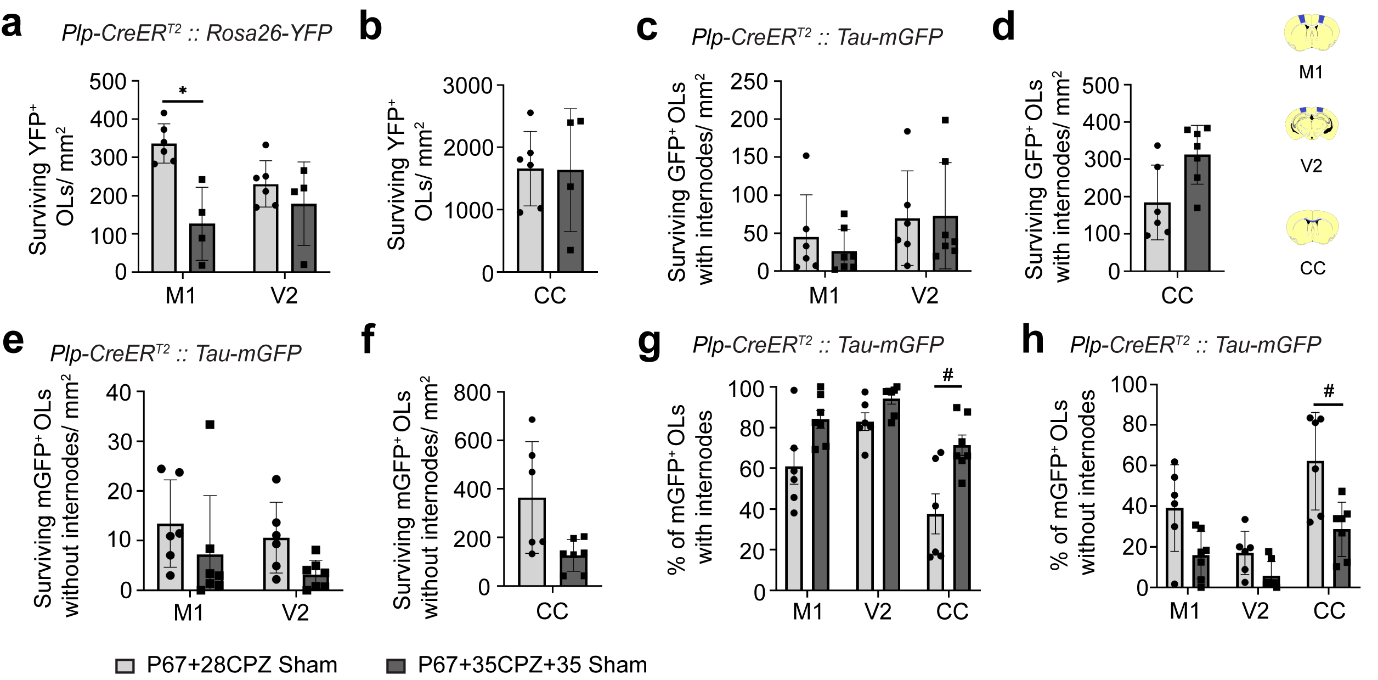


***Figure S8: OL loss occurs more slowly in M1 than V2 or the CC with CPZ feeding, but more of the surviving CC OLs transition from being completely demyelinated to supporting new internodes after CPZ withdrawal.***

**a-b.** The density of surviving YFP^+^ OLs in M1, V2 (**a**) and the CC (**b**) of P67+28CPZ *Plp-CreER^T2^ :: Rosa26-YFP* sham mice (n=6) or P67+35CPZ+35 *Plp-CreER^T2^ :: Rosa26-YFP* sham mice (n=4). Repeated measures two-way ANOVA with Geisser-Greenhouse correction: treatment F (1, 8) = 0.24, p = 0.64; region F (1.0, 8.1) = 39.98, p = 0.0002; interaction F (2, 16) = 0.15, p = 0.86. **c-d.** The density of surviving mGFP^+^ OLs with internodes in M1, V2 (**c**) and the CC (**d**) of P67+28CPZ *Plp-CreER^T2^ :: Tau-mGFP* sham mice (n=6) and P67+35CPZ+35 *Plp-CreER^T2^ :: Tau-mGFP* sham mice (n=7). Repeated measures two-way ANOVA with Geisser-Greenhouse correction: treatment F (1, 11) = 1.34, p = 0.271; region F (1.26, 13.86) = 83.6, p < 0.0001; interaction F (2, 22) = 10.14, p = 0.0008. **e-f.** The density of surviving mGFP^+^ OLs with internodes in M1, V2 (**e**) and the CC (**f**) of P67+28CPZ *Plp-CreER^T2^ :: Tau-mGFP* sham mice (n=6) and P67+35CPZ+35 *Plp-CreER^T2^ :: Tau-mGFP* sham mice (n=7). Repeated measures two-way ANOVA with Geisser-Greenhouse correction: treatment F (1, 11) = 7.91, p = 0.017; region F (1.004, 11.05) = 27.11, p = 0.0003; interaction F (2, 22) = 6.48, p = 0.006. **g.** The proportion (%) of surviving mGFP^+^ OLs with internodes in M1, V2 and the CC of P67+28CPZ *Plp-CreER^T2^ :: Tau-mGFP* sham mice (n=6) and P67+35CPZ+35 *Plp-CreER^T2^ :: Tau-mGFP* sham mice (n=7). Repeated measures two-way ANOVA with Geisser-Greenhouse correction: treatment F (1, 11) = 10.92, p = 0.007; region F (1.79, 19.67) = 28.31, p < 0.0001; interaction F (2, 22) = 3.047, p = 0.068. **h.** The proportion (%) of surviving mGFP^+^ OLs without internodes in M1, V2 and the CC of P67+28CPZ *Plp-CreER^T2^ :: Tau-mGFP* sham mice and P67+35CPZ+35 *Plp-CreER^T2^ :: Tau-mGFP* sham mice. Repeated measures two-way ANOVA with Geisser-Greenhouse correction: treatment F (1, 11) = 10.92, p = 0.007; region F (1.788, 19.67) = 28.31, p < 0.0001; interaction F (2, 22) = 3.047, p = 0.068. Schematics of coronal brain sections show the analysed regions in blue i.e. M1 (~ Bregma +0.5), V2 (~ Bregma -2.5) and CC underlying M1 (~ Bregma +0.5). Data are presented as mean ± SD. Bonferroni post-test: *p < 0.05, # p = 0.051.
